# Decoding the Post-translational Modification Crosstalk: Functional Implications of Phosphorylation, Acetylation, and Methylation

**DOI:** 10.1021/acs.jpcb.5c08630

**Published:** 2026-02-06

**Authors:** Xuyang Qin, Shikha Nangia

**Affiliations:** Department of Biomedical and Chemical Engineering, 2029 Syracuse University, Syracuse, New York 13244, United States

## Abstract

Post-translational modifications (PTMs) such as phosphorylation, acetylation, and methylation critically expand proteome function by regulating protein structure and interactions. Hydropathy changes serve as a main driving force; however, a quantitative, mechanistic understanding of how their distinct chemical changes alter local protein hydropathy remains limited. To bridge this gap, we extend the Protocol for Assigning a Residue’s Character on a Hydropathy (PARCH) scale, a residue-level hydropathy scale, to systematically evaluate PTM-induced physicochemical changes. By applying this method, we quantify the effect and magnitude of hydropathy shifts at modification sites and map how these perturbations influence the local protein environment. Our analysis reveals that phosphorylation exerts a strong, consistent hydrophilic effect, significantly increasing PARCH values due to the introduction of a large, charged phosphate group. In contrast, *N*-lysine acetylation, which neutralizes charge, shows context-dependent effects, predominantly increasing the hydrophobicity but occasionally enhancing the local hydrophilicity. Methylation presents the most complex signature, with no uniform trend, where increased side chain bulk can paradoxically increase water exposure despite the modification’s nonpolar nature. This study establishes the PARCH scale as a powerful quantitative tool for deciphering how PTMs regulate the local hydropathy landscape of proteins, providing a predictive foundation for understanding their structural, hydropathy, and functional consequences.

## Graphical abstract

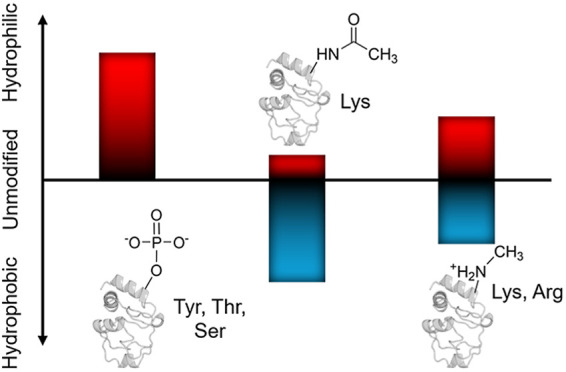

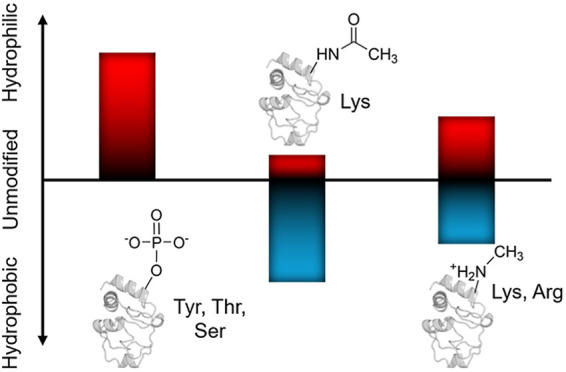

## Introduction

Post-translational modification (PTM) is the covalent alteration of a protein’s residues after its synthesis. This involves the enzymatic or spontaneous addition of a functional group or the chemical modification of an existing one, typically occurring on the amino acid side chains or at the protein’s terminus. The functional complexity of the proteome is critically expanded by PTMs, which act as dynamic chemical regulators of the protein structure, activity, and interaction networks. Among these, phosphorylation, acetylation, and methylation are three of the most abundant and functionally diverse regulatory mechanisms (Figure 1).

**Figure 1:**
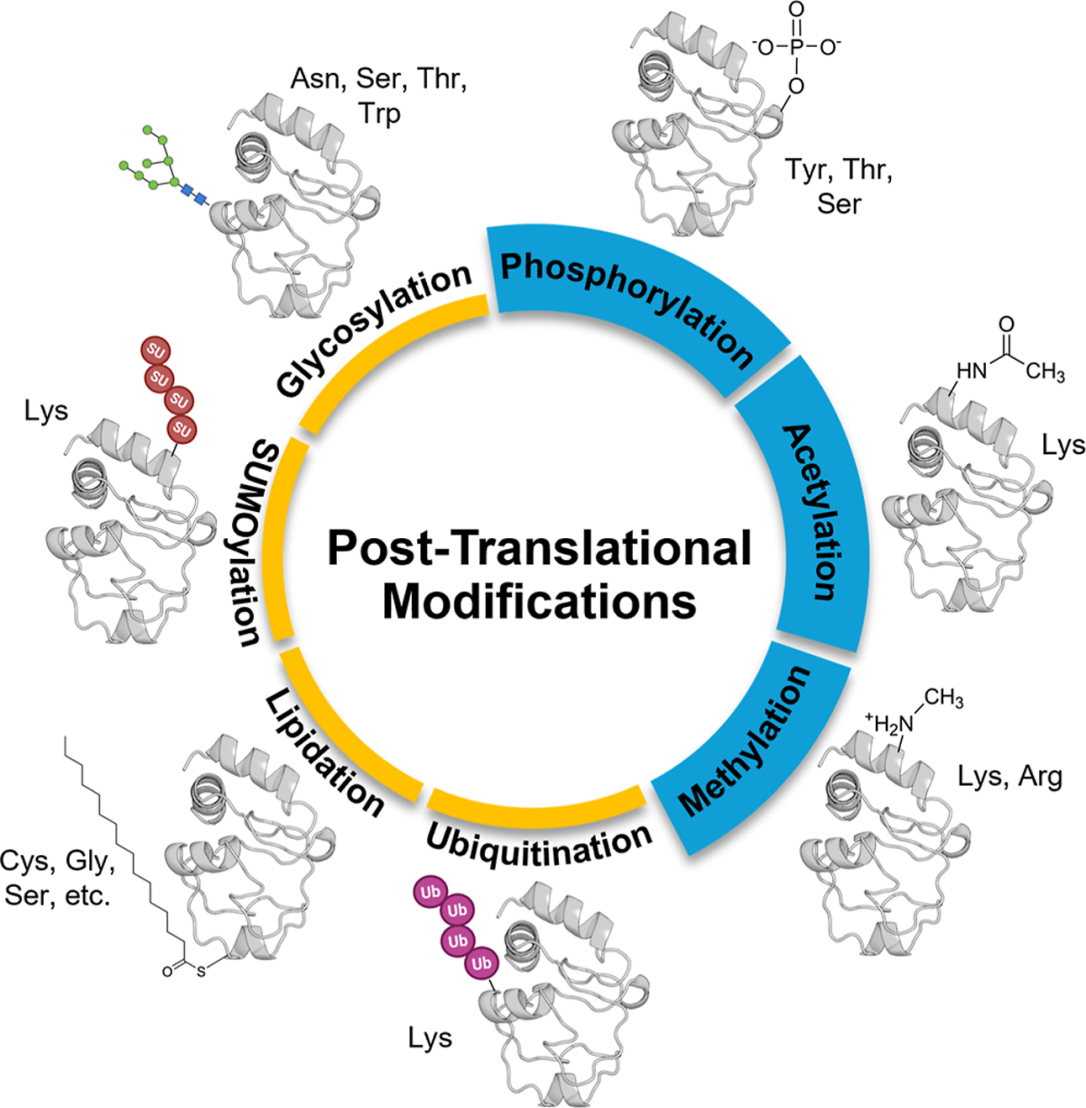
Overview of common post-translational modifications (PTMs). Phosphorylation, acetylation, and methylation are the three of the most prevalent PTMs that modify specific amino acid residues to reshape protein’s structure, interactions, and function.

Phosphorylation adds a charged phosphate group to serine (Ser or S), threonine (Thr or T), or tyrosine (Tyr or Y) residues, serves as a ubiquitous molecular switch in signal transduction,^1^ often inducing conformational changes and adjusting docking sites for effector proteins.^1–3^ According to phosphorylation databases, over two-thirds of the human proteins have already been experimentally confirmed to be phosphorylated.^1^ Acetylation involves the addition of an acetyl group to lysine amine (or protein *N*-terminal), which neutralizes the residue’s positive charge, known to regulate interactions in the protein complex.^4–7^ Methylation is the addition of one to three methyl groups to lysine or arginine side chains, providing a more subtle chemical change that modulates side-chain volume and hydrophobicity, playing a central role in epigenetic regulation^8^ and protein–protein interactions.^9^

Despite their widespread impact on proteins, a fundamental and quantitative understanding of their effect on protein surface properties has remained less explored. The core of protein folding, stability, and molecular recognition lies in hydropathy,^10,11^ which is the thermodynamic driver that governs residue solvation and packing. Conventional hydrophobicity scales fixed static values to canonical amino acids but cannot account for the topographic and chemical changes imposed by PTMs. The introduction of a highly charged phosphate, the neutralization of lysine by an acetyl group, or the incremental addition of nonpolar methyl groups each reforms the residue’s polarity, charge, and volume. This transformation can locally alter the hydropathy character of a protein surface, reshaping the topography of existing binding pockets or creating new interaction sites. A systematic, scale-based framework to quantify chemical and topographic shifts is therefore critical to move from the descriptive identification of PTM sites to the predictive modeling of their structural and functional impacts.

To bridge this gap, we extended the Protocol for Assigning a Residue’s Character on a Hydropathy (PARCH) scale^12^—a residue-level quantitative hydropathy profiler originally developed for unmodified proteins. In the PARCH framework, a uniform shell of water is placed around the protein and the system is gradually heated to promote the “evaporation” of water molecules from the protein surface. The extent to which each residue retains or loses water during this simulated annealing process intrinsically captures the interplay between nanoscale topography and local chemistry. Each residue receives a PARCH value ranging from 0 to 10, where 0 indicates highly hydrophobic, poorly hydrated sites and 10 indicates strongly hydrophilic, water-retaining sites.

Building on this foundation, we generated a PARCH database of more than 1,000 proteins, revealing how residue identity, local geometry, and surrounding nanoscale features collectively determine hydropathy.^12^ We further demonstrated that the method is robust across multiple water models (TIP3P, TIP4P, TIP4P-Ew, TIP5P), yielding consistent hydropathy trends independent of solvent parametrization.^13^ Most recently, we extended PARCH to nucleic acids and nucleic-acid–protein complexes,^14^ enabling hydropathy assignment to both nucleotide backbones and bases and revealing striking contrasts—for example, the high hydrophilicity of phosphate–sugar backbones relative to nucleobases. Together, these advances establish PARCH as a versatile, chemistry- and topology-aware hydropathy scale applicable across proteins, DNA, RNA, and their complexes.

In this work, we further extend the PARCH framework to quantify the hydropathy of phosphorylated, acetylated, and monomethylated amino acid residues, enabling residue-level comparisons before and after PTMs. This capability provides mechanistic insight into how PTMs modulate local hydropathy and, in turn, reprogram protein structure, dynamics, and function. By aligning amino acid sequences and comparing PARCH values at modification sites, we can (i) quantify the magnitude and direction of hydropathy changes; (ii) map how these perturbations propagate across the local nanoscale environment and alter topography; and (iii) correlate distinct hydropathy signatures with underlying biochemical and signaling mechanisms. Collectively, this approach will establish PARCH as a powerful foundation for interpreting PTM effects and decoding the hydropathy-based logic of cellular signaling networks.

## Methods

### Protein Modification and Equilibration

A representative set of phosphorylation (ph), acetylation (ac), and monomethylation (me) PTM sites was identified for this study through a combination of literature curation and the PhosphoSitePlus database,^53^ an integrated resource that catalogs experimentally validated and computationally predicted post-translational modifications. Corresponding protein structures were obtained from the Protein Data Bank (in PDB format), and the associated PDB IDs along with the PTM site information are summarized in Table 1. For simulations, each protein system was prepared with the CHARMM36m force field^54^ via the CHARMM-GUI server^55,56^ to incorporate the PTMs and solvate in TIP3P water^54,57^ with 0.15 M NaCl. Both unmodified and modified versions of each protein were prepared. All simulations were performed with GROMACS 2023.2.^58^ The systems were first energy-minimized using the steepest-descent algorithm with a force tolerance of 1 × 10^3^ kJ mol^–1^ nm^–1^. Equilibrium simulations were carried out under isothermal–isochoric (NVT) and isothermal–isobaric (NPT) ensembles for 1 ns each at 300 K with a 2 fs time step. During NVT equilibration, positional restraints were applied to all protein heavy atoms using a force constant of 400 kJ mol^–1^ nm^–2^, which was reduced to 40 kJ mol^–1^ nm^–2^ during NPT equilibration to promote gradual structural relaxation. Electrostatic and van der Waals interactions were treated using a 1.2 nm cutoff, while long-range electrostatics were calculated with the Particle-Mesh Ewald (PME)^59,60^ method. Temperature was controlled using the *V*-rescale^61^ thermostat with a 1.0 ps coupling constant. During NPT equilibration, isotropic pressure was maintained at 1 bar using the Berendsen^62^ barostat with a 5.0 ps coupling constant and a compressibility of 4.5 × 10^–5^ bar^–1^. All hydrogen-involving bonds were constrained using the LINCS algorithm.^63^ Following equilibration, a 2 ns production run was carried out under NPT conditions using the Parrinello–Rahman^64^ barostat and without position restraints to capture short-time scale conformational changes. To enable a direct comparison between modified and unmodified systems, the production length was chosen to reflect the localized and fast-relaxing nature of the hydration-based observables analyzed here. PARCH probes nanoscale water–residue coordination rather than slow, global conformational rearrangements, which could confound interpretation if extensively sampled.

**Table 1:** Proteins and PTMs Studied

PTMs	protein	PDB ID	PTM sites
phosphorylation (ph)	FGFR1	4UWY^15^	Y653, Y654 ^16^
	CDK2	1JST^17^	Y15 ^18^
			T14 ^18^
	4E-BP2	2MX4 ^3^	T37, T46, S65, T70, S83 ^19^
	KRas G12D	4DSO^20^	Y32 ^21^
	MKK4	3ALO^22^	S257, T261 ^23^
	CYCS (human)	2N9I^24^	Y48 ^25^
	CYCS (rat)	5C0Z^26^	T58 ^27^
	NCAP	6M3M^28^	T76, S78, S79, S105, T166 ^29^
acetylation (ac)	CDK1	4Y72 ^30^	K33 ^31^
	Adk	1AKE^32^	K136, K141, K145, K157, K192, K211 ^33^
	Fmt	2FMT^34^	K45, K46 ^33^
	YaaA	5CAJ^35^	K55 ^33^
	GapA	1S7C	K61, K124, K132, K138, K184, K192, K249 ^36^
	PfkA	1PFK^37^	K317 ^38^
	Eda	1EUA^39^	K24, K25 ^38^
	PgmA	1E59 ^40^	K17, K85, K99, K141, K145 ^38^
	TalB	4S2C^41^	K4, K50, K187, K250, K301, K308 ^38^
monomethylation (me)	p53	1DT7 ^42^	K382 ^43^
	NF-κB p65	1NFI^44^	K310 ^45^
	Histone3	1ID3 ^46^	K79 ^47^
	NDKA	1JXV^48^	R6, R58 ^49^
	CYB5B	3NER^50^	K39 ^51^
	PP1G	1JK7 ^52^	R36 ^49^

### Simulation Setup for PARCH Simulations

After equilibration, the unmodified and modified structures of each protein were aligned, and PARCH analysis was performed following our established protocol.^13^ For post-translationally modified residues, all atoms of the modified residue were treated explicitly and consistently with the standard PARCH protocol. Briefly, each protein was embedded within an explicit water shell of thickness *d*_shell_ = 0.415 nm and restrained by using a force constant of 10^4^ kJ mol^–1^ nm^–2^. Hydrated counterions were placed based at a distance *d*_ion_ = 3 nm from the protein surface to balance the net charge of the system, and the ion-box boundary distance *d*_*b*_ was set as 3 nm. The counterions were also position-restrained during annealing to ensure stability and reproducibility of the PARCH calculations. The systems were energy-minimized using the steepest descent algorithm with 10^3^ kJ mol^–1^ force tolerance and then simulated in the *NVT* ensemble condition with increasing temperatures (300 to 800 K at a rate of 1 K/10 ps) using the annealing protocol in GROMACS. Each system was performed in quintuplicates to ensure that PARCH values were sampled well. The cutoff for water molecules contacting a residue *d*_water_ was set at 0.315 nm to quantify and scale the PARCH values. In the neighborhood within the 0.3 nm cutoff around the PTM sites, each residue’s PARCH value was compared between the unmodified and modified proteins. Based on empirical observations, a PARCH difference greater than ±0.2 was considered indicative of a significant hydropathy change at that residue and counted as a meaningful alteration in the local environment.

## Results and Discussion

### Phosphorylation Acts as a Powerful Hydrophilic Switch at the PTM Site and in Its Neighborhood

Phosphorylation adds the phosphate group carrying a −2 charge to the Ser, Thr, and Tyr residues, which significantly increases their polarity, which markedly increases their polarity and capacity for hydrogen bonding. Figure 2 summarizes how this chemical change translates into quantitative shifts in residue-level and local hydropathy for a representative set of phosphorylation sites across several proteins. In Figure 2, the colored bars report the change in the PARCH value (ΔPV) at each modified residue, while the gray bars show the cumulative ΔPV within a 3 Å neighborhood, capturing how the microenvironment responds to the modification.

**Figure 2:**
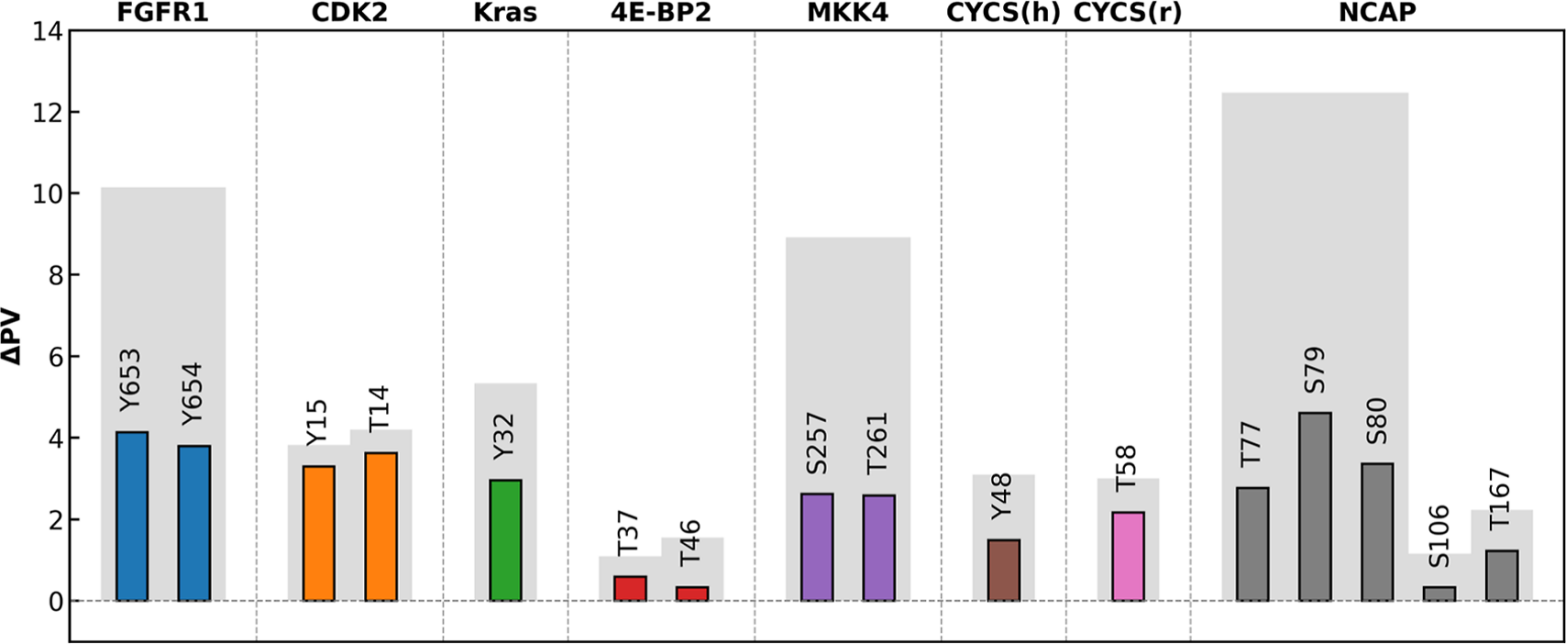
Site-specific and local hydropathy changes (ΔPV) upon phosphorylation. Colored bars represent the change in PARCH values at the labeled phosphorylation sites. Gray bars indicate the cumulative PARCH value change within a 3 Å neighborhood of each site.

Across all systems analyzed, phosphorylation consistently increased the hydrophilicity of the modified residue, with PARCH increases ranging from 0.33 to 4.13 units. Sites such as Y653 in FGFR1 or S79 in NCAP show particularly large ΔPV, reflecting a substantial enhancement of local water affinity at the modification site. Importantly, the surrounding residues do not remain unaffected: the gray bars reveal sizable positive shifts in the neighborhood PARCH values, demonstrating that phosphorylation propagates a hydropathy change beyond the modified residue and renders the local surface patch more hydrophilic. Such localized “hydropathy remodeling” can affect the hydrogen-bonding. The results further suggest that, in signaling proteins, such hydropathy changes can be key drivers of conformational rearrangements or altered binding affinities, thereby enabling functional activation or inhibition. The impact of this phosphorylation-induced hydropathy tuning is illustrated in the case studies discussed below, where we examine how specific structural contexts translate hydropathy changes to functional outcomes.

The **FGFR1 (Fibroblast Growth Factor Receptor 1)** kinase plays a pivotal role in regulating cell fate, proliferation, differentiation, and homeostasis.^16,65^ Its activation is tightly controlled by the phosphorylation of two highly conserved residues, Y653 and Y654, located in the activation loop (Figure 3). These residues function as molecular switches that determine the conformational and catalytic state of the kinase. Biochemical studies have shown that phosphorylation of Y653 alone increases catalytic activity by 50- to 100-fold, while subsequent phosphorylation of Y654 enhances activity by an additional 500- to 1000-fold. These findings underscore their essential role in full receptor activation.^65^

**Figure 3:**
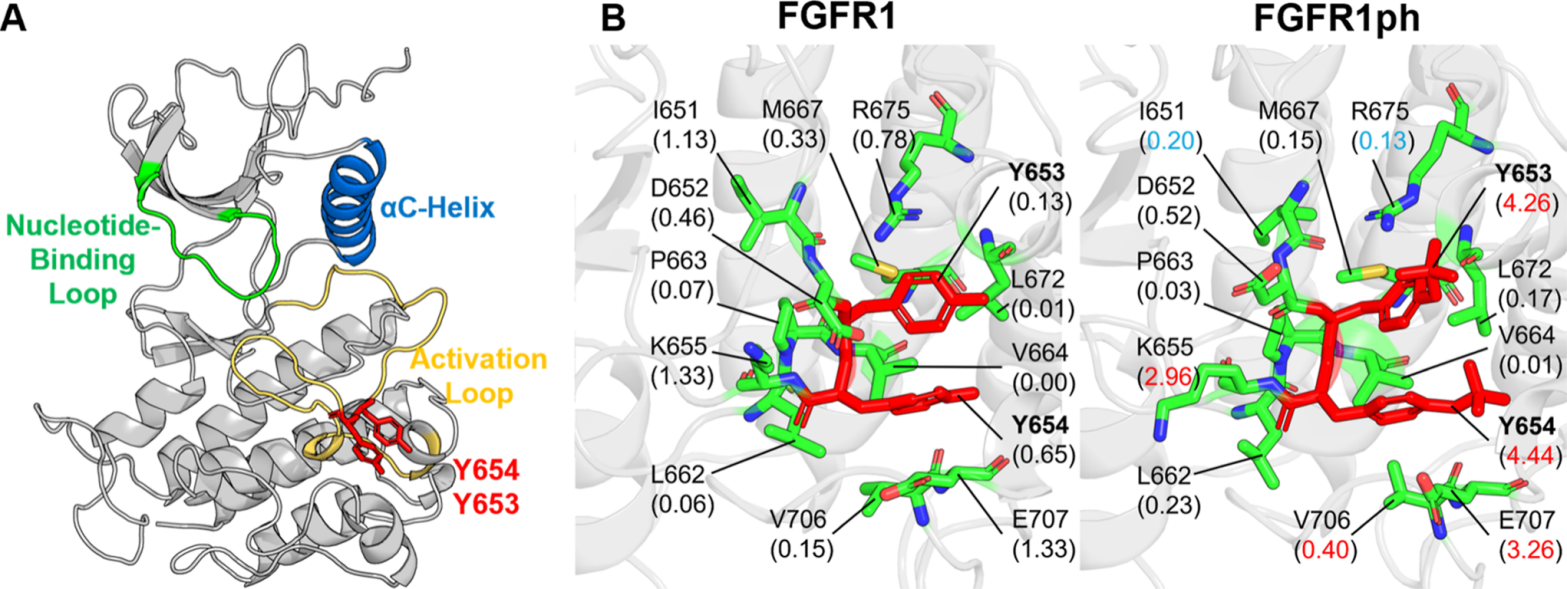
Comparative PARCH analysis of FGFR1 near phosphorylation sites Y653 and Y654. (A) Structure of unphosphorylated FGFR1 kinase with key structural elements. (B) PARCH values of unmodified and phosphorylated states. Residues are labeled with their identities and PARCH values in parentheses. The red value indicates a PARCH value increase >0.2 upon phosphorylation; blue indicates a decrease >0.2.

The biophysical effect of phosphorylation arises from the introduction of two negatively charged phosphate groups into the activation loop, creating both steric hindrance and electrostatic repulsion that force the loop outward. This conformational transition stabilizes the open, catalytically competent state of FGFR1, aligning critical active-site residues and clearing the substrate-binding cleft for the efficient phosphorylation of downstream targets. Earlier structural analyses also identified Y653 and Y654 as indispensable components of the autophosphorylation cascade required for full receptor activation.^66^

Figure 3A highlights the key regulatory elements of the FGFR1 kinase domain, including the nucleotide-binding loop (green), the alpha C helix (blue), and the activation loop (yellow), with Y653 and Y654 shown in red. In the unmodified state (Figure 3B, left panel), these residues are embedded in a compact hydrophobic environment, stabilized by contacts with the surrounding side chains, such as L662, V664, and M667. Upon phosphorylation (Figure 3B, right panel), the introduction of two negatively charged phosphate groups generates strong steric and electrostatic repulsion, driving the activation loop outward and exposing it to the solvent.

This structural transition is captured quantitatively by the PARCH hydropathy analysis, which reveals substantial increases in water affinity for both Y653 and Y654, with ΔPV values of 2.90 and 3.26, respectively. Several neighboring residues, including residues K655, V706, and E707, also show elevated PARCH values, demonstrating that phosphorylation induces both site-specific and neighborhood-level hydropathy remodeling. A few residues, such as I651 and R675, show slight decreases in hydrophilicity, reflecting local rearrangements within the activation loop.

Heterogeneous PARCH responses among residues neighboring a PTM site do not reflect simple sequence proximity but instead arise from the nanoscale structural organization of the local environment. Post-translational modification reshapes charge distribution and steric constraints, perturbing hydrogen-bonding networks, side-chain orientations, and solvent accessibility in a spatially anisotropic manner. Consequently, residues that are geometrically and dynamically coupled to the reorganized hydration network exhibit pronounced changes in water affinity, whereas others in sequence proximity may remain largely unaffected.

For example, in Figure 3B, K655 shows a marked increase in PARCH because its side chain becomes favorably oriented to cooperatively retain interfacial water following phosphorylation-induced restructuring of the activation loop. In contrast, P663, despite being nearby in sequence, is weakly coupled to the modified hydration environment and shows little change in PARCH. These results demonstrate that PTM-induced hydropathy remodeling is governed by local packing, side-chain orientation, and hydration-network coupling rather than by a monotonic distance-dependent effect. Accordingly, PARCH reports site-specific, directionally resolved hydration coordination associated with functional nanoscale reorganization.

Together, these structural and hydropathy changes support a unified mechanistic view: phosphorylation at Y653 and Y654 stabilizes the open, catalytically competent conformation of FGFR1 and reshapes the hydration landscape of the activation loop. The increased local hydrophilicity enhances loop flexibility, promotes solvent exposure, and facilitates the alignment of key catalytic residues. These features are essential for efficient substrate access and kinase activity. This combined structural and PARCH-based analysis shows that phosphorylation-induced hydropathy tuning directly contributes to the functional activation of FGFR1.

**CDK2 (cyclin-dependent kinase 2)** is a central regulator of cell cycle progression, governing the onset and fidelity of DNA replication.^18,67^ Phosphorylation of T14 and Y15, two residues located within the ATP-binding cleft, functions as a key inhibitory mechanism that prevents premature CDK2 activation.^18^ Structural visualization in Figure 4A shows that both residues sit adjacent to the bound ATP molecule, positioning them to directly influence nucleotide access. Our PARCH analysis (Figures 4B,C) reveals that phosphorylation produces very large increases in local hydrophilicity: Y15 increases from 0.36 to 3.65 and T14 increases from 0.51 to 4.13, representing approximately an order of magnitude change at each site. These substantial shifts, highlighted in red font in Figure 4, could result in substantial steric and electrostatic barriers around the nucleotide-binding pocket, causing the disruption of the ATP orientation and blocking substrate binding. The phosphorylated residues also influence the physicochemical environment of nearby positions such as K33 and R36 further reshaping the catalytic cleft. Together, these effects corroborate with the notions that the CDK2 is in an inactive state until the inhibitory phosphates are removed. This phospho-switch, as demonstrated by the PARCH values, appears to act as a critical checkpoint that halts cell cycle progression in response to DNA damage or replication stress.^18^ It has been reported that dysregulation of T14 and Y15 phosphorylation is observed in cancer and highlights the essential role of this control mechanism in maintaining cell cycle fidelity.^18^

**Figure 4:**
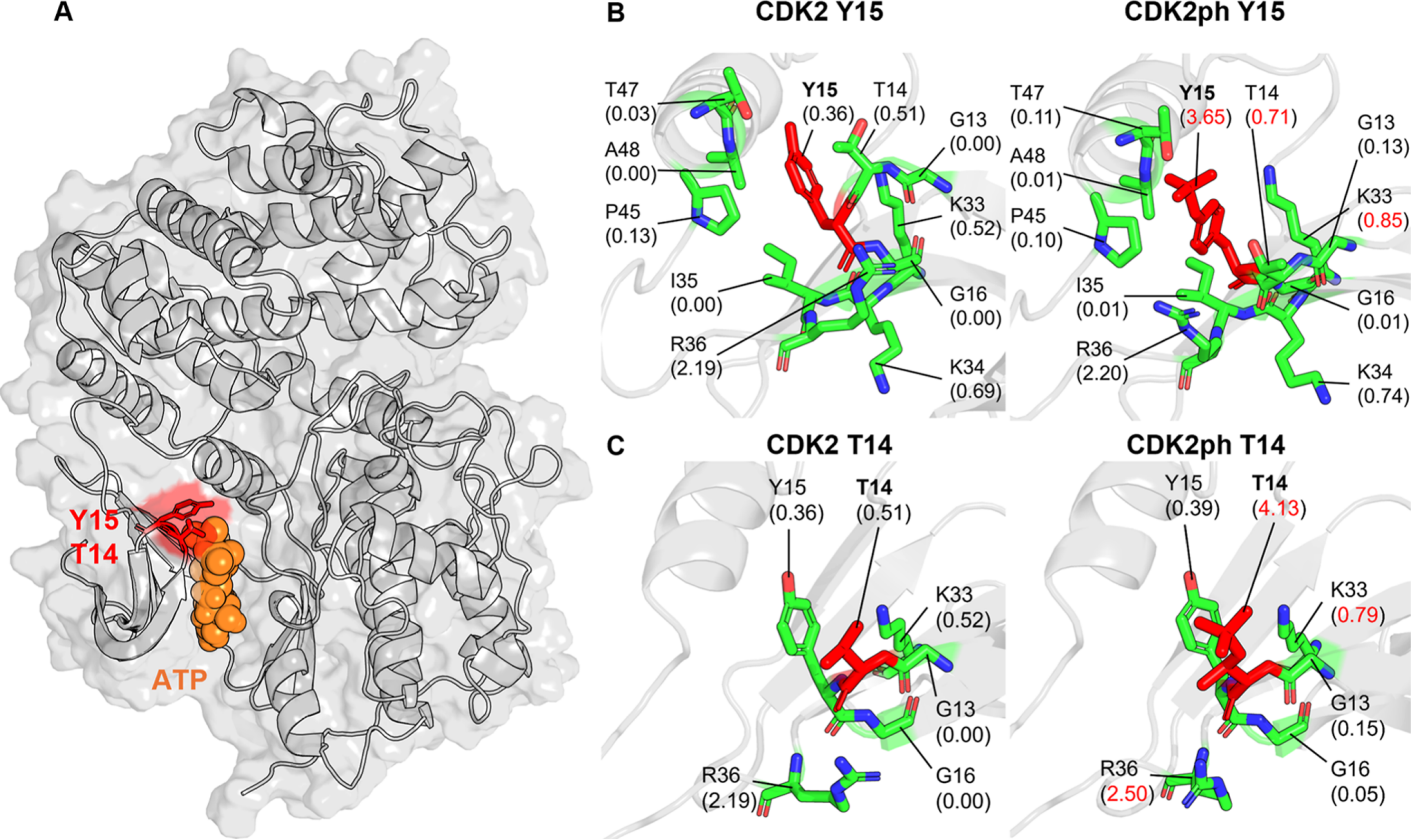
Comparative PARCH analysis of CDK2 near phosphorylation sites T14 or Y15. (**A**) Structure overview of the unphosphorylated CDK2 in complex with ATP, with T14 and Y15 shown in the ATP-binding cleft. (**B,C**) PARCH analysis of unmodified and phosphorylated states for (B) Y15 and (C) T14. Residues are labeled with their identities and PARCH values in parentheses. The red value indicates a PARCH value increase >0.2 upon phosphorylation; blue indicates a decrease >0.2.

**KRas** is a critical GTPase (guanosine triphosphatase) protein that functions as a molecular switch, regulating key cellular processes like growth and survival, whose dysregulation performs as a major driver in many cancers.^68^ The phosphorylation at Y32 acts as a molecular brake, silencing the KRas signaling. The steric bulk introduced by the phosphorylation directly impedes GTP binding (Figure 5A). Meanwhile, it is reported that the phosphorylation of Y32 acts as an inactivator, by disrupting the local conformation of KRas’s flexible switch 1 as more flexible and dynamic.^21^ As shown in Figure 5, phosphorylation dramatically increases the PARCH value at Y32 from 1.71 to 4.47, with increases observed for nearby residues D12, G13, D33, and Q61. This shift increases local hydrophilicity to make the switch 1 region more exposed to the solvent environment, and it disrupts the GTP-binding pocket to impair the downstream signal transmission as reported in other work.^21,69^

**Figure 5:**
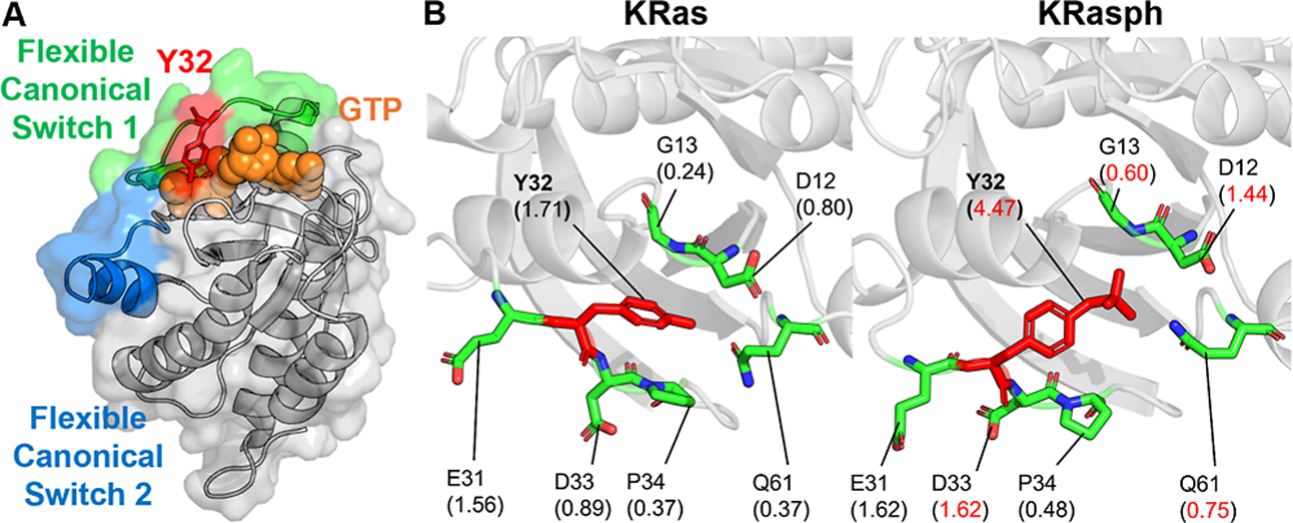
Comparative PARCH analysis of KRas near phosphorylation site Y32. (A) Structure overview of the unphosphorylated KRas in complex with GTP, with Y32 shown in the flexible canonical switch 1. (B) PARCH analysis of unmodified and phosphorylated states. Residues are labeled with their identities and PARCH values in parentheses. The red value indicates a PARCH value increase >0.2 upon phosphorylation; the blue value indicates a decrease >0.2.

The impact of phosphorylation on four additional proteins is shown in the Supporting Information (Figures S1–S7). Figure S8 summarizes the effects of phosphorylation on local hydrophilicity using PARCH values for residues surrounding each modification site. A paired, nonparametric Wilcoxon signed-rank test was used to evaluate whether phosphorylation leads to statistically significant changes in PARCH across the set of nearby residues for each protein. For each protein and modification site, individual residue-level changes are displayed as paired points connected by lines, which visualize how the PARCH value shifts upon phosphorylation. Proteins such as CDK2 (T14 and Y15), 4EBP2 (T46), MKK4, and NCAP (T167 and T77/T79/S80) exhibit significant increases in hydrophilicity, consistent with phosphorylation introducing a strong polar character into the local environment. However, in some cases, such as FGFR1, Karas, CYCS human, and CYCS rat, only a few residues change their hydropathy without a substantial shift relative to neighboring residues, which are labeled as not significant (ns) in Figure S8. Overall, the PARCH scale analysis indicates that phosphorylation acts as a strong hydrophilic switch at modification sites and at neighboring residues, modulating the protein function.

### Acetylation’s Hydrophobic Influence on the Site and Its Neighborhood Is Highly Site-Specific

*N*-Lysine acetylation neutralizes the positive charge on the lysine side chain while adding an acetyl group to the amino group, a modification often assumed to uniformly increase the residue hydrophobicity. However, our PARCH analysis of nine proteins shows that acetylation induces site-specific changes in hydropathy rather than a simple global shift (Figure 6). While many lysine residues and their neighboring positions become more hydrophobic, several sites exhibit pronounced increases in PARCH values or broader cumulative effects across the surrounding region.

**Figure 6:**
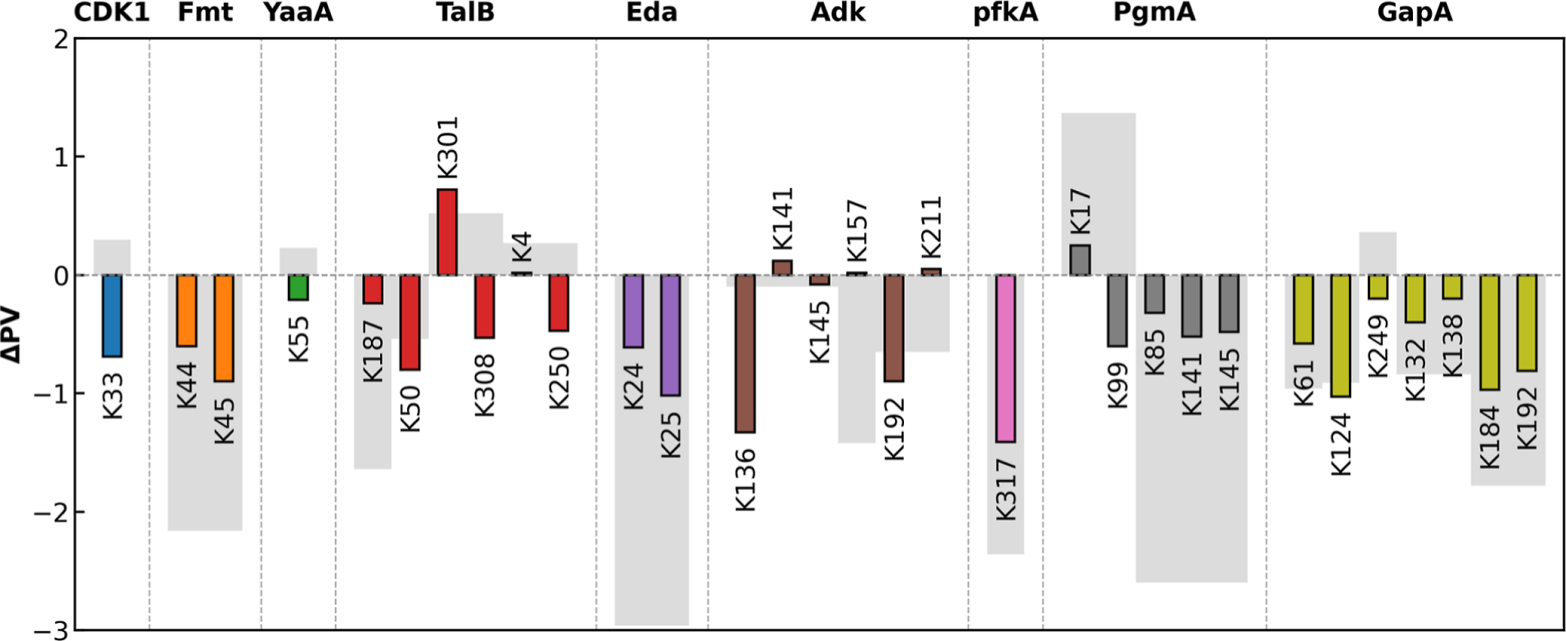
Site-specific and local hydropathy changes upon lysine acetylation. Colored bars represent the change in PARCH value at the labeled acetylation sites. Gray bars indicate the cumulative PARCH value change more than 0.2 within a 3 Å neighborhood of each site.

These hydropathy changes arise from two key factors: (1) the intrinsic polarity of the acetyl group, which modulates local hydrophilicity even as the lysine charge is neutralized, and (2) the specific conformational adjustments of the acetylated side chain within its local structural environment. Together, these effects reshape the local hydrophilic landscape in ways that can influence protein–protein and protein–ligand interactions. Specific examples of these consequences are described next.

**CDK1 (Cyclin-Dependent Kinase 1)** is a crucial master regulator of the eukaryotic cell cycle, essential for mitosis, but its overactivity or deregulation also significantly contributes to tumor initiation and progression in many cancers, acting as an oncogene by promoting uncontrolled cell division and survival.^67^ Acetylation of K33, a conserved residue within the ATP-binding kinase domain (Figure 7A), directly suppresses CDK1’s catalytic activity and downstream signaling. Mechanistically, K33 acetylation disrupts the CDK1–STAT3 axis, leading to reduced STAT3 phosphorylation and diminished expression of stemness-associated genes. This modification adds steric bulk that obstructs ATP binding (Figure 7B) while simultaneously neutralizing the positive charge of K33. K33 is also a key binding site for small-molecule inhibitors, such as the flavonoid fisetin.

**Figure 7:**
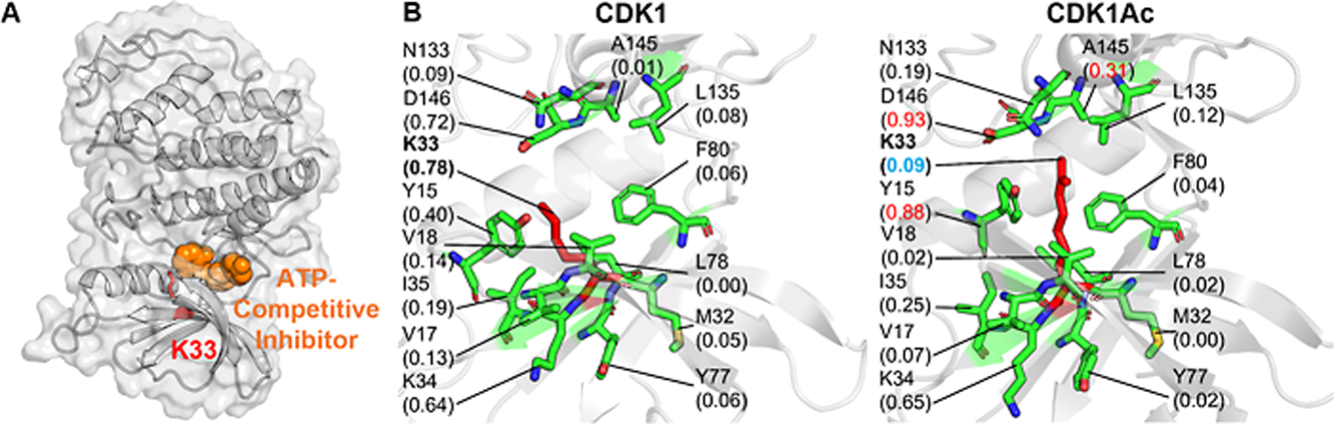
Comparative PARCH analysis of CDK1 near acetylation site Y32. (A) Structure overview of the unacetylated CDK1 in complex with an ATP-competitive inhibitor, where K33 located on the ATP-binding kinase domain. (B) PARCH analysis of unmodified and acetylated states. Residues are labeled with their identities and PARCH values in parentheses. The red value indicates a PARCH value increase >0.2 upon acetylation; the blue value indicates a decrease >0.2.

Our PARCH analysis showed that K33 became more hydrophobic upon acetylation. The increased side-chain volume allows acetylated K33 to form new contacts with residues D146 and A145, interactions that are not present in the unmodified protein. These additional contacts produce localized increases in PARCH values within the surrounding region.

Fmt (methionyl-tRNA formyltransferase) is a bacterial enzyme that catalyzes the initial step in protein synthesis by producing formyl-methionyl-tRNA (fMet-tRNA), a molecule required to initiate nearly all protein synthesis in bacterial cells.^34^ Structurally, a key loop (Loop 1) within the catalytic domain directly contacts the tRNA substrate^34^ (Figure 8A). Though the functional consequences of Fmt acetylation are not fully studied, acetylation at K44 and K45, located on Loop 1, has been experimentally confirmed.^33^ Their placement suggests a potential role in modulating the enzyme’s catalytic function. As shown in Figure 8B, the PARCH scale shows that acetylation leads to a drop in PARCH values at K44 and K45, as well as at neighboring residues P39, G41, and L46. Since Loop 1 interacts with the charged backbones and polar nucleobases of tRNA, the elimination of positive charge and reduction in local hydrophilicity at K44 and K45 may impede tRNA binding and hinder catalytic activity.

**Figure 8:**
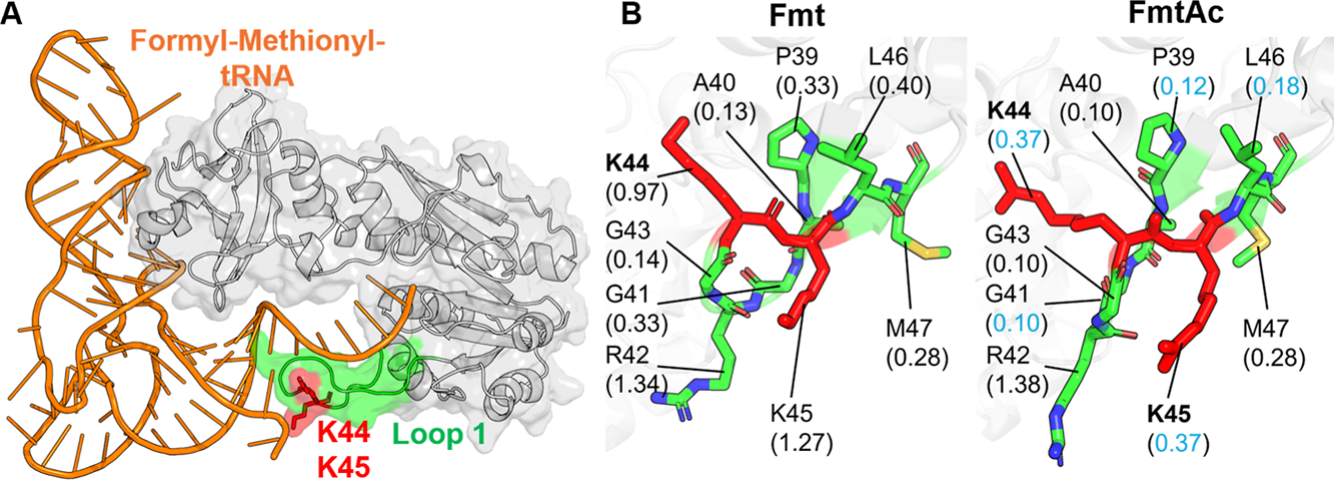
Comparative PARCH analysis of Fmt near acetylation sites K44 and K45. (A) Structure overview of the unacetylated Fmt in complex with a formyl-methionyl-tRNA, where K44 and K45 located on the tRNA-binding loop 1. (B) PARCH analysis of unmodified and acetylated states. Residues are labeled with their identities and PARCH values in parentheses. The red value indicates a PARCH value increase >0.2 upon acetylation; the blue value indicates a decrease >0.2.

**YaaA** is a bacterial protein in the DUF328 family that binds DNA and plays a key role in protecting cells from hydrogen peroxide-induced oxidative stress.^33,35^ Structural analysis revealed a novel protein fold containing a classical helix-hairpin-helix (HhH) DNA-binding motif (Figure 9A), a feature common to many DNA repair enzymes and essential for YaaA’s ability to interact with diverse DNA structures. K55, an experimentally confirmed acetylation site located on the HhH motif,^70^ is predicted to modulate YaaA’s function within the bacterial stress response.^35^

**Figure 9:**
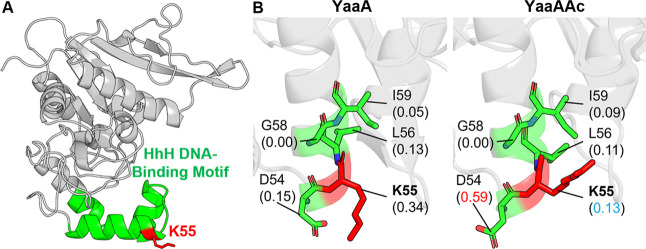
Comparative PARCH analysis of YaaA near acetylation site K55. (A) Structure overview of the unacetylated YaaA, where K55 located on the helix-hairpin-helix (HhH) DNA-binding motif. (B) PARCH analysis of unmodified and acetylated states. Residues are labeled with their identities and PARCH values in parentheses. The red value indicates a PARCH value increase >0.2 upon acetylation; the blue value indicates a decrease >0.2.

PARCH analysis (Figure 9B) shows that acetylation at K55 increases the local hydrophobicity, reflected by a decrease in the PARCH value from 0.34 to 0.13. Because K55 lies near the protein surface with relatively few neighboring residues, its modification produces only a localized effect. The small increase in the PARCH value observed for the adjacent residue D54 likely arises from conformational adjustments in both D54 and K55 following acetylation.

Across all proteins examined, the comparative PARCH analyses (Figures S9−S23) reveal that lysine acetylation generates highly site-specific hydropathy changes rather than a uniform shift in hydrophobicity. Each acetylation site, whether in TalB, Eda, Adk, PfkA, PgmA, or GapA, produces a distinct local response, with some residues and neighboring regions showing marked increases in hydrophobicity. These patterns demonstrate that the hydropathy outcome of acetylation is determined by the local structural and chemical environment, creating a protein-specific and site-specific redistribution of hydrophobic and hydrophilic character.

### Monomethylation Alters Local Hydropathy in a Residue-Dependent Manner within Structured Protein Regions

Monomethylation involves the addition of a single methyl group to the side chains of lysine and arginine residues. In contrast to acetylation, which typically neutralizes charge, monomethylation preserves the positive charge on the side chain while modifying its steric and hydrophobic properties. While methylation frequently occurs within intrinsically disordered regions (IDRs) of proteins, such as histone tails and RNA-binding proteins, the inherent flexibility of IDRs leads to rapid structural variation. This conformational dynamic complicates direct comparisons of PARCH values derived from largely disparate structures. Therefore, we focused our investigation on methylation sites located within ordered protein regions.

Introducing a methyl group is generally expected to increase the local hydrophobicity. Consistent with this expectation, Figure 10 shows that monomethylation decreases PARCH values at the modification site and surrounding residues for K79 on histone, K19 on CYB5B, and R6 on NDKA. However, the opposite trend occurs at other sites, such as K107 on p53, K310 on NFκB, and R36 on PP1G, where PARCH values instead increase upon monomethylation. These localized increases in hydrophilicity may arise not only from changes in side-chain configuration but also from the redistribution of charge over the larger methylated group, which can alter how water molecules interact with the residue. The functional examples that follow illustrate these diverse methylation-driven influences on local hydropathy.

**Figure 10:**
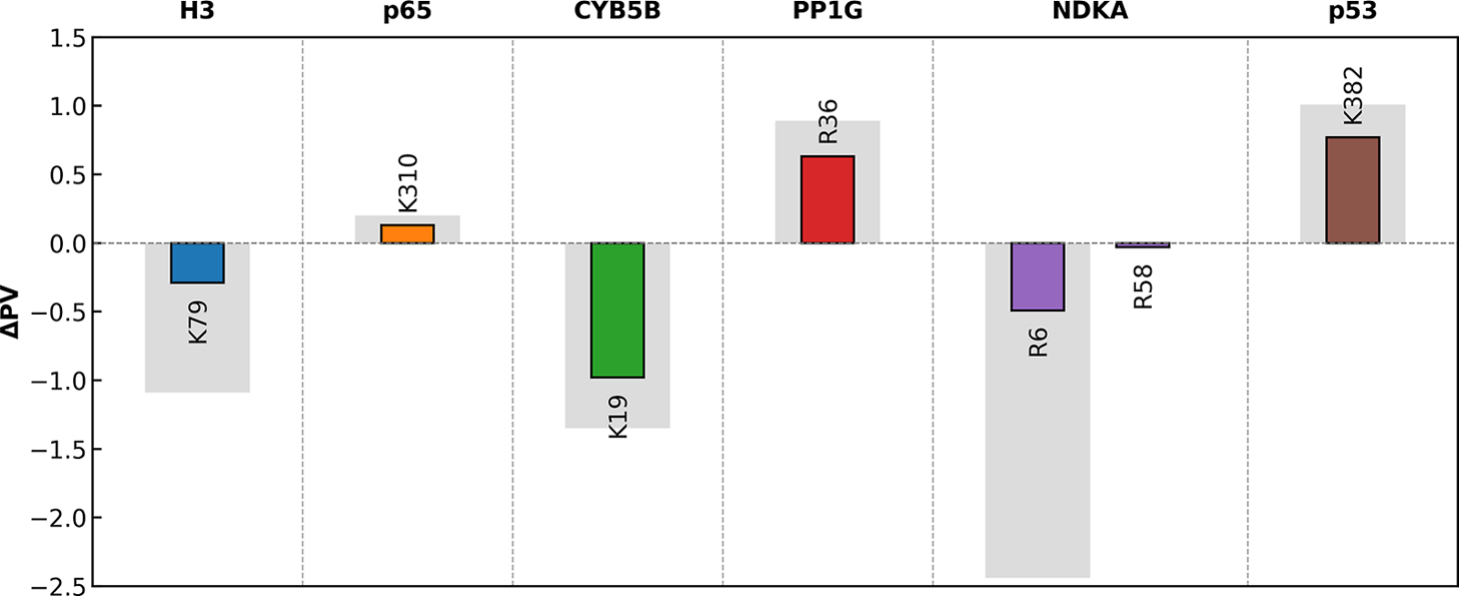
Site-specific and local hydropathy changes upon monomethylation. Colored bars represent the change in PARCH value at the labeled methylation sites. Gray bars indicate the cumulative PARCH value change within a 3 Å neighborhood of each site.

**Histones** are the core protein components of nucleosomes, the fundamental repeating units of chromatin that package and organize DNA within the cell nucleus. Together with post-translational modifications, it regulates all DNA-templated processes, including gene transcription, DNA repair, and chromosome segregation.^47^ Lysine 79 (K79) on Histone H3 is characterized as a methylation site. Unlike the methylations on the flexible histone tails, K79 is located at the core of the histone H3 protein (Figure 11A). Its monomethylation is a foundational mark often associated with actively transcribed genes and serves as a precursor for further methylations.^47^ Methylation prevents the binding of silencing complexes (e.g., SIR) to chromatin, with its dysregulation directly implicated in developmental defects and cancers. As shown in Figure 11B, the hydrophilicity decreases at the K79 site and surrounding residues, which may lead to enhanced stacking between multiple histones and downstream gene expression.

**Figure 11:**
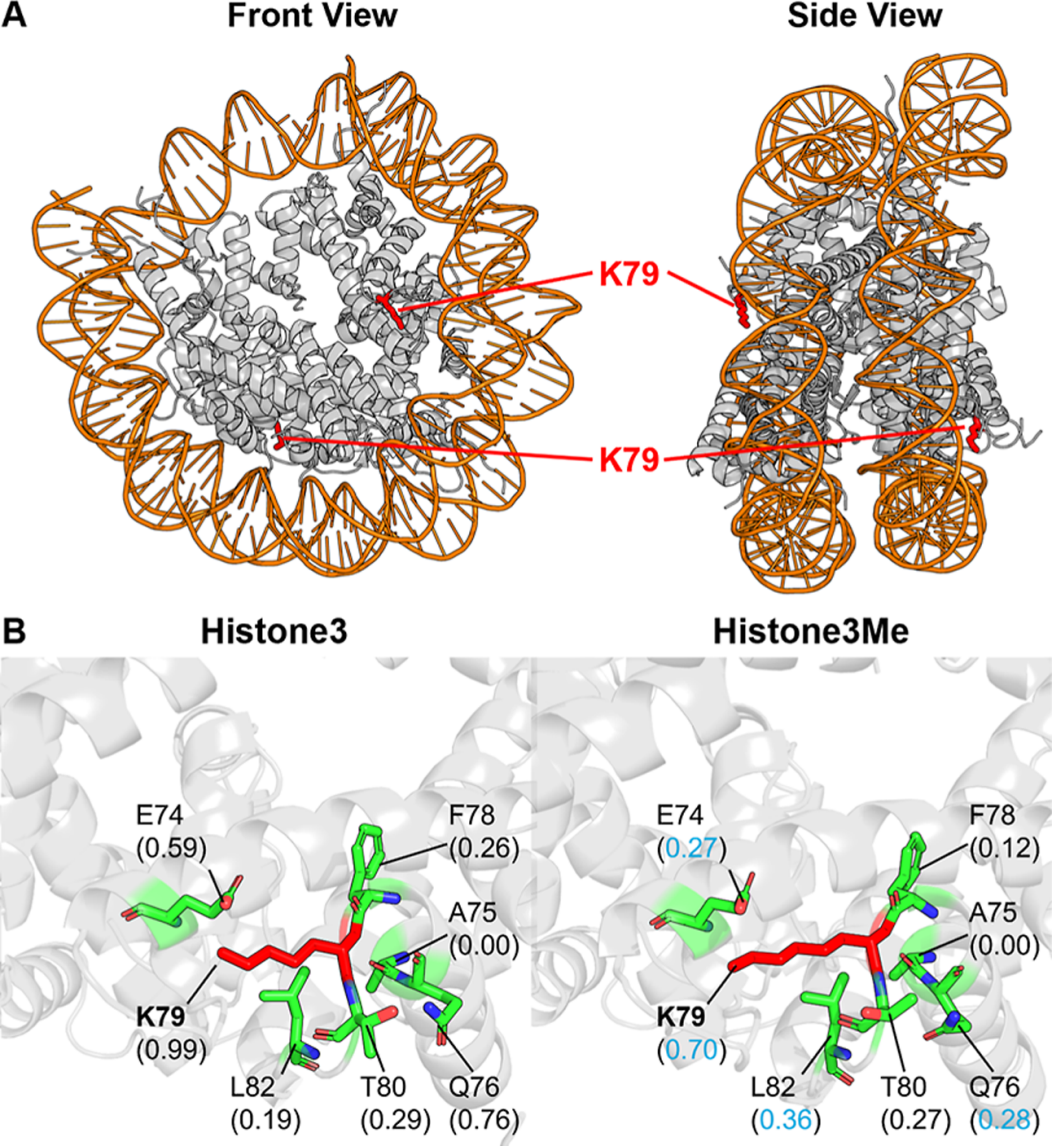
Comparative PARCH analysis of histone near monomethylated site K79 on H3. (A) Structure overview of unmethylated histone in complex with DNA (PDB: 1ID3). (B) PARCH analysis of unmodified and acetylated states. Residues are labeled with their identities and PARCH values in parentheses. A blue value indicates a decrease >0.2 upon methylation.

As a member of **NF-κB (transcription factor nuclear factor κB)**, **p65** acts as a transcription factor in inflammatory and immune responses.^45^ The methylation of lysine 310 (K310) acts as a critical “brake” in preventing inappropriate inflammatory responses and is dysregulated in disease. The monomethylation of K310 leads to the induction of a repressed state of NF-κB target genes through the binding of G9a-like protein.^45^ It facilitates the deposition of the repressive histone methylation mark, thereby enforcing a repressed state on a subset of inflammatory genes and preventing their inappropriate expression. A structure study shows that the K310 nearby region contacts an NF-κB-inhibitor-α (Figure 12A). Based on PARCH analysis (Figure 12B), the local region does not show a major shift in hydrophobicity; specifically, no significant PARCH decrease is observed upon monomethylation. K310 itself shows a slight, nonsignificant PARCH increase. The most notable change is a 0.20 PARCH increase for M313. Given the high solvent exposure of this area, minor hydropathy increases like these may result from additional side-chain volume introduced by the methyl group, rather than a substantial change in local hydrophobicity.

**Figure 12:**
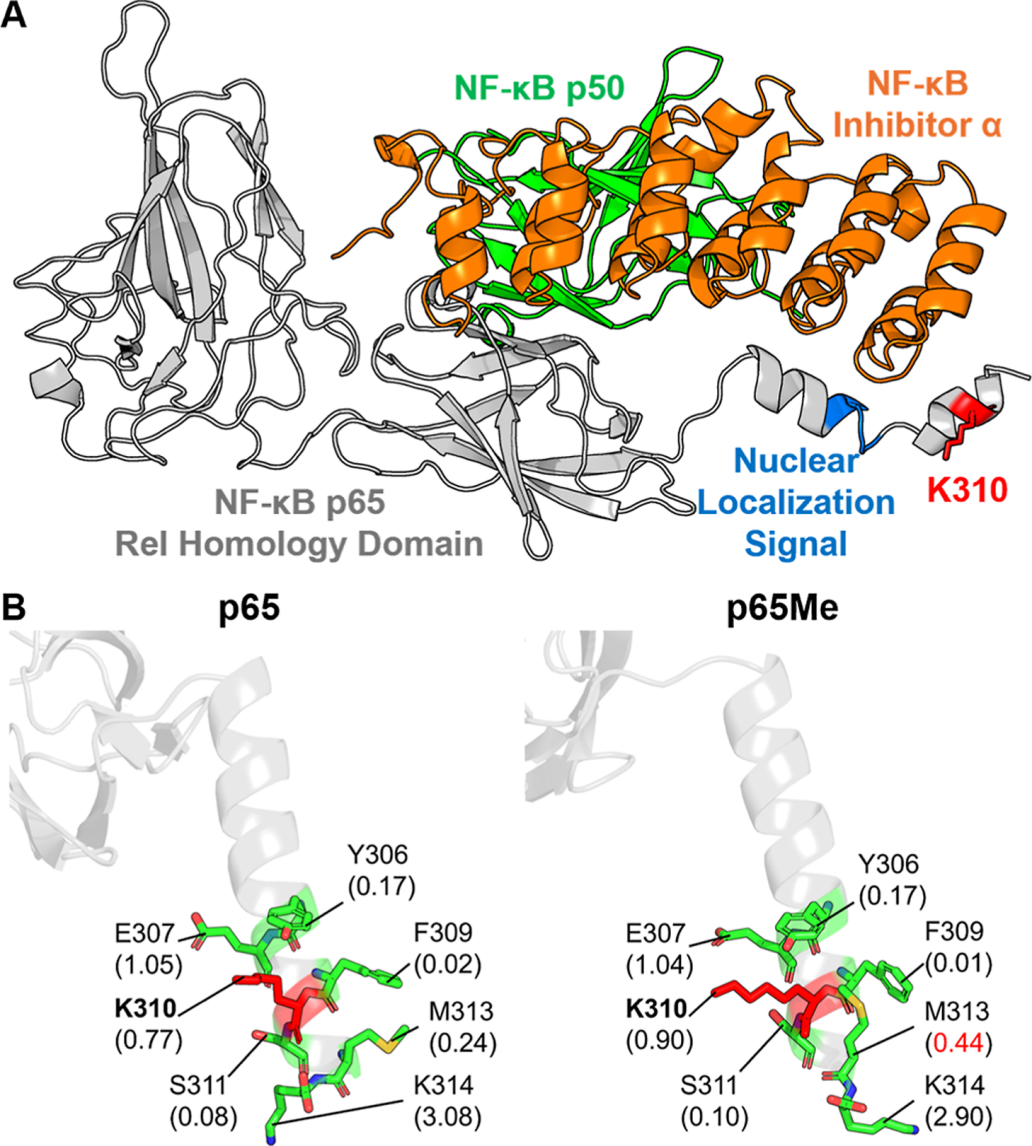
Comparative PARCH analysis of NF-κB p65 near monomethylated site K310. (A) Structure overview of the unmethylated p65 in complex with p50 and an inhibitor protein (PDB: 1NFI). (B) PARCH analysis of unmodified and acetylated states. Residues are labeled with their identities and PARCH values in parentheses. The red value indicates a PARCH value increase >0.2 upon methylation; the blue value indicates a decrease >0.2.

Across all proteins examined, the comparative PARCH analyses in Figures S24−S28 reveal a distinctly site-specific response to monomethylation. Each modification produces localized shifts in hydropathy, sometimes increasing hydrophobicity and sometimes decreasing it, with no uniform directional trend across proteins or even within the same protein. Whether in CYB5B, PP1G, NDKA at two different sites, or p53, monomethylation consistently alters the local chemical environment in a residue-dependent manner, reinforcing that methylation-induced hydropathy changes are highly context-dependent.

## Conclusions

By extending the PARCH scale to analyze post-translational modifications, we were able to evaluate in detail how different PTMs alter hydropathy at both the modified residues and their surrounding protein environments. Our analyses revealed that each type of modification exerts a distinct physicochemical influence. Phosphorylation produces the strongest and most consistent effect: the addition of a large, negatively charged phosphate group substantially increases hydrophilicity, leading to pronounced increases in PARCH values at the site and nearby residues. In contrast, the impact of *N*-lysine acetylation is more dependent on the local structural context. Although most acetylated sites show decreased PARCH values, indicating increased hydrophobicity, several sites display increased hydrophilicity instead. These exceptions likely arise from changes in residue configuration, the steric bulk that alters water accessibility, or polarity contributions from the acetyl group. Monomethylation presents an even more nuanced pattern. Because it preserves the positive charge while enlarging the side chain, the resulting hydropathy shifts do not follow a consistent directional trend. In some cases, the added methyl group enhances hydrophobicity, as expected, while in others, the increased side-chain volume appears to expose the residue to additional water molecules, leading to higher PARCH values. Overall, this study demonstrates that the PARCH scale provides a robust quantitative framework for deciphering how diverse PTMs reshape the local hydropathy landscape of proteins and, in turn, modulate their structural and functional behavior.

## Supplementary Material



## Data Availability

The data set for all structures and computed PARCH values for the protein is available at https://github.com/NangiaLab/PARCH-PTMs. There is no restriction on the use of the data.

## Notes

PARCH analysis of phosphorylation sites on 4E-BP2, MKK4, CYCS, and NCAP proteins, along with a statistical summary of phosphorylation-induced changes; acetylation analyses for TalB, Eda, Adk, pfkA, PgmA, and GapA, covering both individual lysine sites and clusters of modification; and methylation effects on CYB5B, PP1G, NDKA, and p53 (PDF)
