# Supplementary material for: Decoding the Post-translational Modification Crosstalk: Functional Implications of Phosphorylation, Acetylation, and Methylation
Source: J Phys Chem B. 2026 Feb 6;130(7):2144–56. doi: 10.1021/acs.jpcb.5c08630 (PMC12926945; doi:10.1021/acs.jpcb.5c08630)
Supplement: Supplementary file 1 [file jp5c08630_si_001.pdf]

Supporting Information

Decoding the Post-translational Modification Crosstalk: Functional Implications of Phosphorylation, Acetylation, and Methylation

Xuyang Qin and Shikha Nangia*

Department of Biomedical and Chemical Engineering, Syracuse University, Syracuse, NY 13244, USA

*Address for correspondence:
Dr. Shikha Nangia
343 Link Hall
Department of Biomedical and Chemical Engineering
Syracuse University, Syracuse, NY 13244, USA
Phone (315) 443 0571 | Email: snangia@syr.edu
ORCID 0000-0003-1170-8461

Table of Contents

Figure S1. Comparative PARCH analysis of 4E-BP2 near phosphorylation sites T37 and T46. ....S3

Figure S2. Comparative PARCH analysis of MKK4 near phosphorylation sites T257 and T261. ....S4

Figure S3. Comparative PARCH analysis of CYCS(h) near phosphorylation sites Y48. ....S5

Figure S4. Comparative PARCH analysis of CYCS(r) near phosphorylation sites T58. ....S6

Figure S5. Comparative PARCH analysis of NCAP near phosphorylation sites T77, S79 and S80. ....S7

Figure S6. Comparative PARCH analysis of NCAP near phosphorylation sites S106. ....S7

Figure S7. Comparative PARCH analysis of NCAP near phosphorylation sites T167. ....S8

Figure S8. Statistical analysis of phosphorylation-induced PARCH value changes. S9

Figure S9. Comparative PARCH analysis of TalB near acetylation sites K187, K301 and K308. ....S10

Figure S10. Comparative PARCH analysis of TalB near acetylation sites K50. ....S10

Figure S11. Comparative PARCH analysis of TalB near acetylation sites K4 and K250. ....S11

Figure S12. Comparative PARCH analysis of TalB near acetylation site K50. ....S11

Figure S13. Comparative PARCH analysis of Eda near acetylation sites K24 and K25. ....S12

Figure S14. Comparative PARCH analysis of Adk near acetylation sites K136, K141 and K145. ....S13

Figure S15. Comparative PARCH analysis of Adk near acetylation site K157. ....S13

Figure S16. Comparative PARCH analysis of Adk near acetylation sites K192 andK211. ....S14

Figure S17. Comparative PARCH analysis of pfkA near acetylation site K317. ....S15

Figure S18. Comparative PARCH analysis of PgmA near acetylation sites K17 and K99. ....S16

Figure S19. Comparative PARCH analysis of PgmA near acetylation sites K85, K141 and K145. ......S16

Figure S20. Comparative PARCH analysis of GapA near acetylation site K61. ....S17

Figure S21. Comparative PARCH analysis of GapA near acetylation site K124. ....S17

Figure S22. Comparative PARCH analysis of GapA near acetylation sites K132 and K138. ....S18

Figure S23. Comparative PARCH analysis of GapA near acetylation sites K184 and K192. ....S18

Figure S24. Comparative PARCH analysis of CYB5B near monomethylation site K382. ....S19

Figure S25. Comparative PARCH analysis of PP1G near monomethylation site R36. ....S20

Figure S26. Comparative PARCH analysis of NDKA near monomethylation site R6. ....S21

Figure S27. Comparative PARCH analysis of NDKA near monomethylation site R58. ....S21

Figure S28. Comparative PARCH analysis of p53 near monomethylation site K382. ....S22

Figure S1. Comparative PARCH analysis of 4E-BP2 near phosphorylation sites T37 and T46.

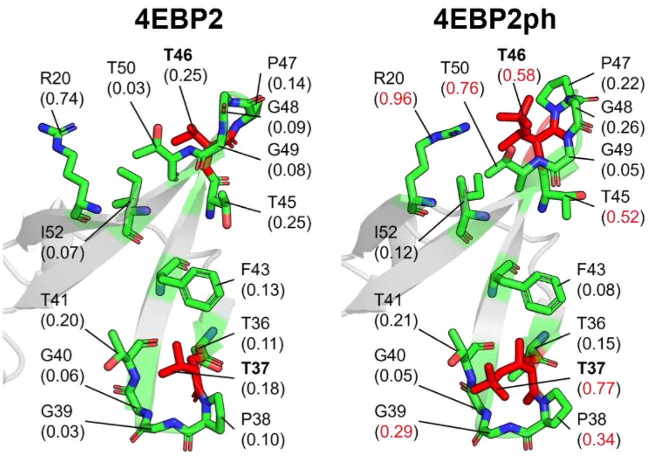

PARCH values of unmodified (left) and phosphorylated (right) states. Residues are labeled with their identities and PARCH values in parentheses. Red value indicates a PARCH value increase > 0.2 upon phosphorylation; blue indicates a decrease > 0.2.

Figure S2. Comparative PARCH analysis of MKK4 near phosphorylation sites T257 and T261.

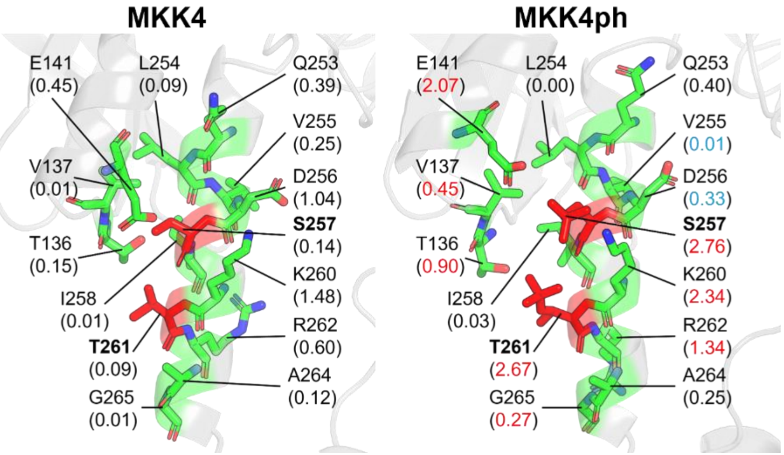

PARCH values of unmodified (left) and phosphorylated (right) states. Residues are labeled with their identities and PARCH values in parentheses. Red value indicates a PARCH value increase > 0.2 upon phosphorylation; blue indicates a decrease > 0.2.

Figure S3. Comparative PARCH analysis of CYCS(h) near phosphorylation sites Y48.

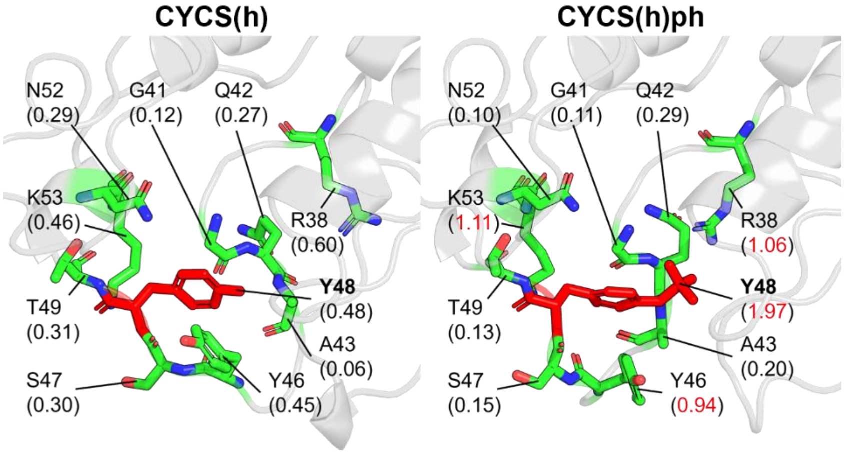

PARCH values of unmodified (left) and phosphorylated (right) states. Residues are labeled with their identities and PARCH values in parentheses. Red value indicates a PARCH value increase > 0.2 upon phosphorylation; blue indicates a decrease > 0.2.

Figure S4. Comparative PARCH analysis of CYCS(r) near phosphorylation sites T58.

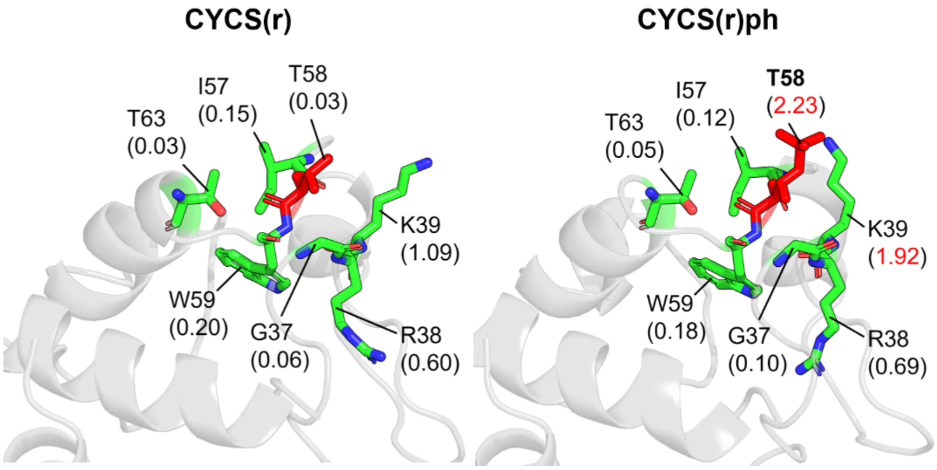

PARCH values of unmodified (left) and phosphorylated (right) states. Residues are labeled with their identities and PARCH values in parentheses. Red value indicates a PARCH value increase > 0.2 upon phosphorylation; blue indicates a decrease > 0.2.

Figure S5. Comparative PARCH analysis of NCAP near phosphorylation sites T77, S79 and S80.

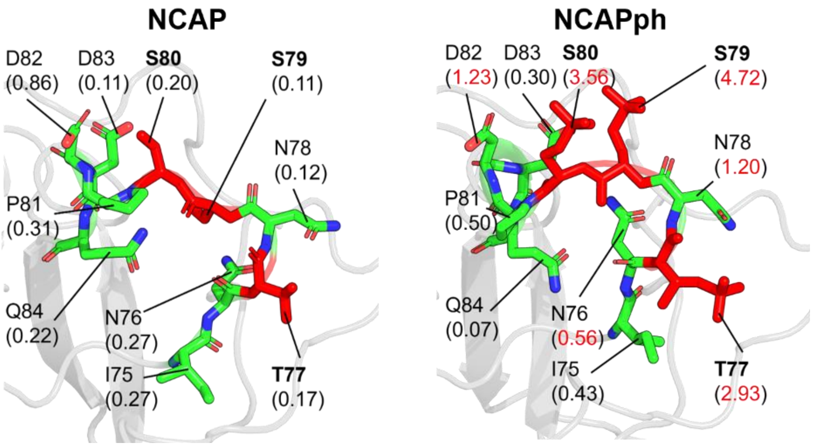

PARCH values of unmodified (left) and phosphorylated (right) states. Residues are labeled with their identities and PARCH values in parentheses. Red value indicates a PARCH value increase > 0.2 upon phosphorylation; blue indicates a decrease > 0.2.

Figure S6. Comparative PARCH analysis of NCAP near phosphorylation sites S106.

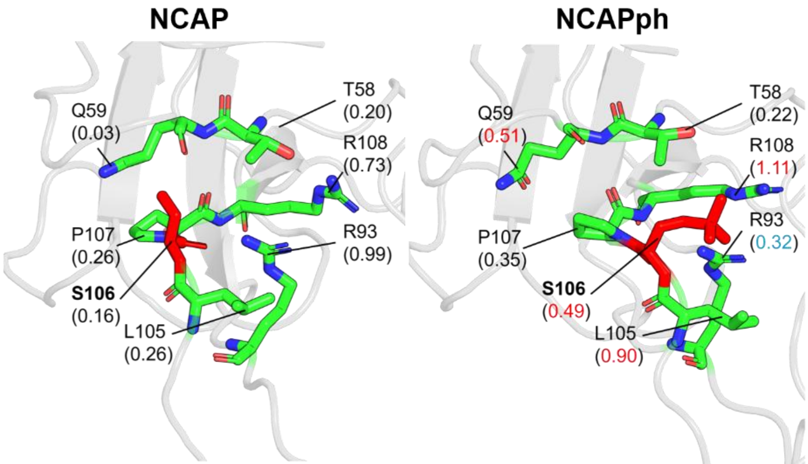

PARCH values of unmodified (left) and phosphorylated (right) states. Residues are labeled with their identities and PARCH values in parentheses. Red value indicates a PARCH value increase > 0.2 upon phosphorylation; blue indicates a decrease > 0.2

Figure S7. Comparative PARCH analysis of NCAP near phosphorylation sites T167.

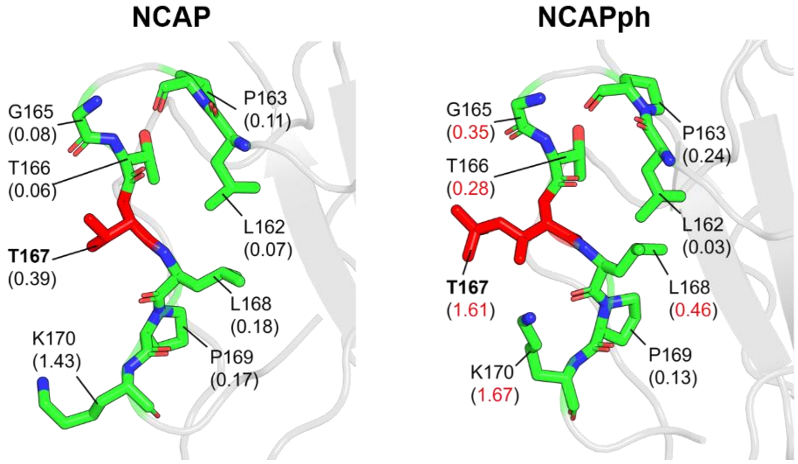

PARCH values of unmodified (left) and phosphorylated (right) states. Residues are labeled with their identities and PARCH values in parentheses. Red value indicates a PARCH value increase > 0.2 upon phosphorylation; blue indicates a decrease > 0.2.

Figure S8. Statistical analysis of phosphorylation-induced PARCH value changes.

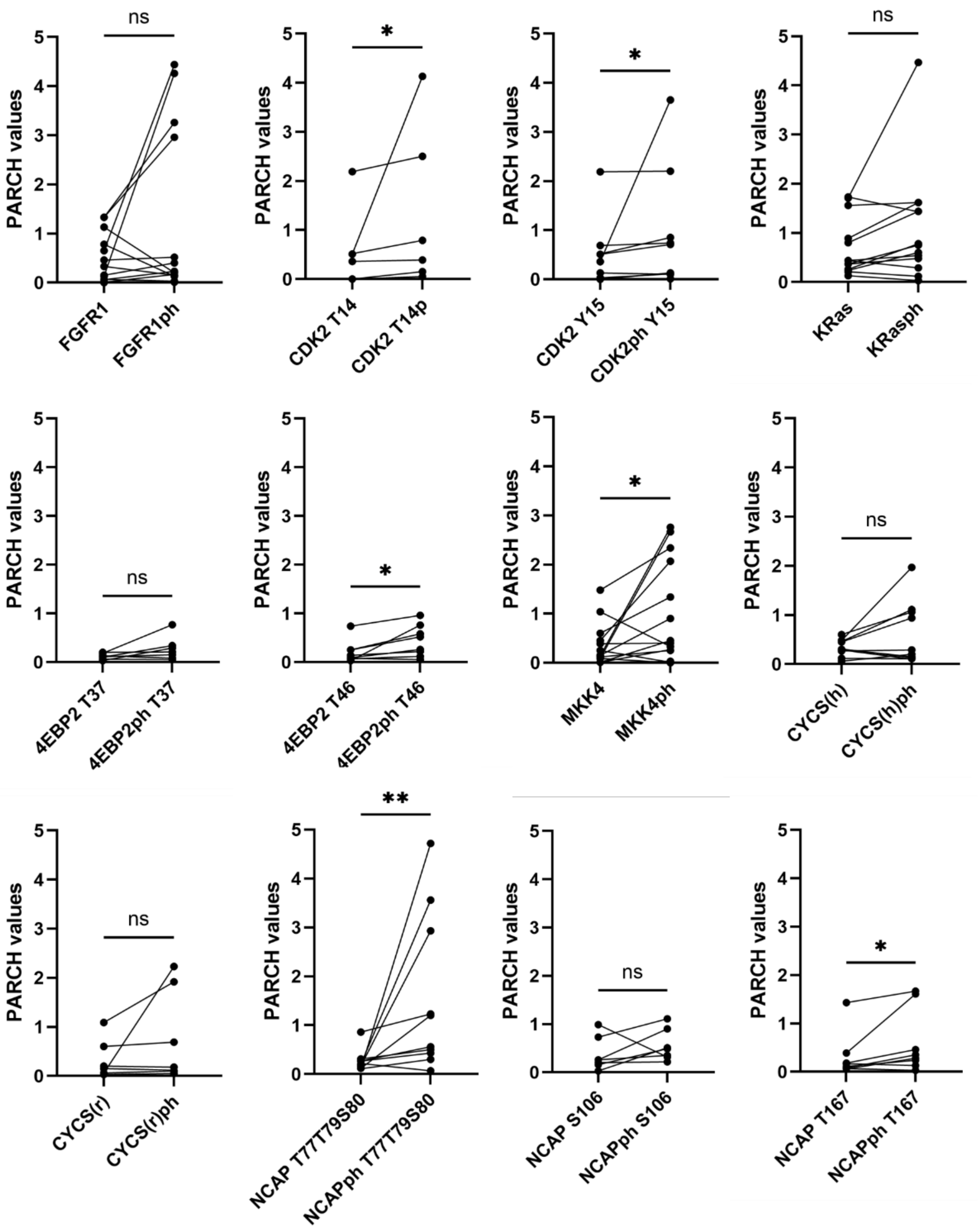

A paired, non-parametric Wilcoxon signed-rank test was applied to quantify the significance of changes for residues near the modification sites. The significance levels are indicated as: ns ($p > 0.05$), * ($p \leq 0.05$), ** ($p \leq 0.01$), and *** ($p \leq 0.001$). Individual residue changes are visualized with dashes connecting unmodified and phosphorylated PARCH values.

Figure S9. Comparative PARCH analysis of TalB near acetylation sites K187, K301 and K308.

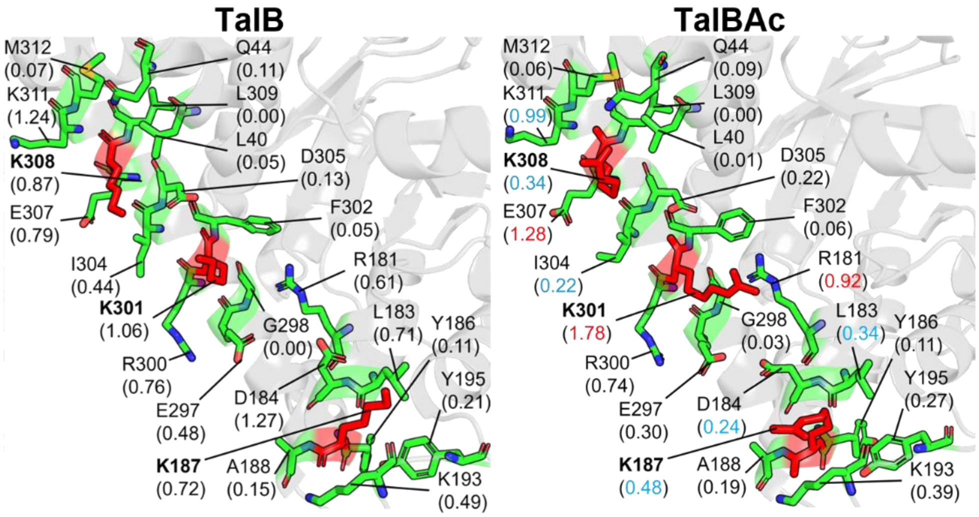

PARCH values of unmodified (left) and acetylated (right) states. Residues are labeled with their identities and PARCH values in parentheses. Red value indicates a PARCH value increase > 0.2 upon acetylation; blue indicates a decrease > 0.2.

Figure S10. Comparative PARCH analysis of TalB near acetylation sites K50.

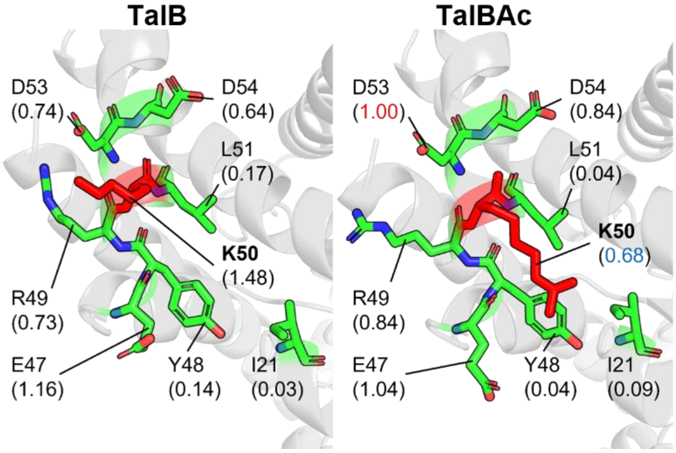

PARCH values of unmodified (left) and acetylated (right) states. Residues are labeled with their identities and PARCH values in parentheses. Red value indicates a PARCH value increase > 0.2 upon acetylation; blue indicates a decrease > 0.2.

Figure S11. Comparative PARCH analysis of TalB near acetylation sites K4 and K250.

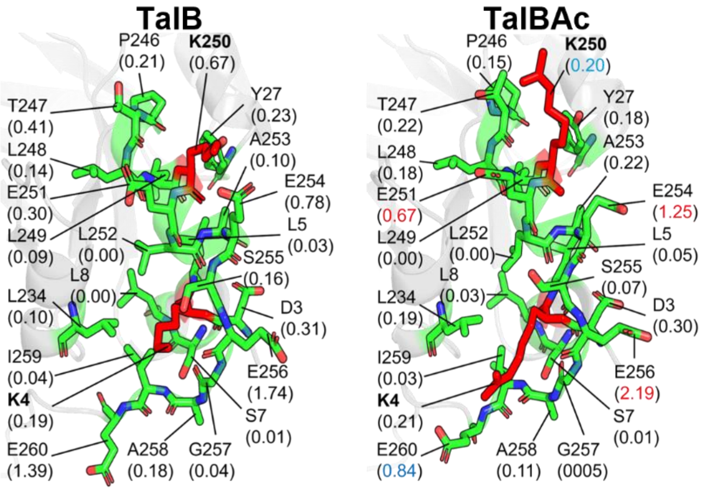

PARCH values of unmodified (left) and acetylated (right) states. Residues are labeled with their identities and PARCH values in parentheses. Red value indicates a PARCH value increase > 0.2 upon acetylation; blue indicates a decrease > 0.2.

Figure S12. Comparative PARCH analysis of TalB near acetylation site K50.

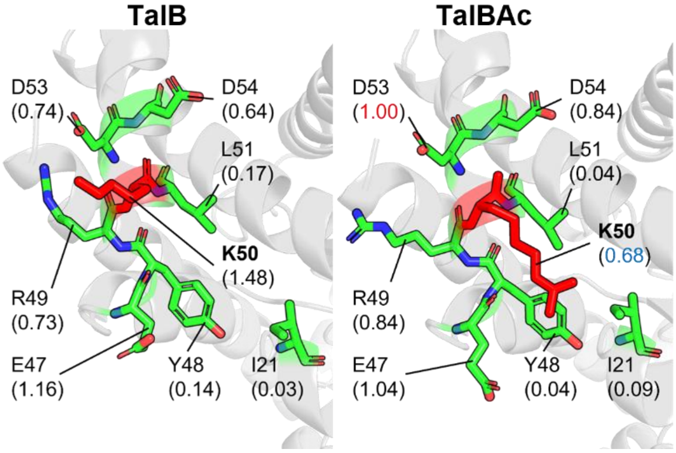

PARCH values of unmodified (left) and acetylated (right) states. Residues are labeled with their identities and PARCH values in parentheses. Red value indicates a PARCH value increase > 0.2 upon acetylation; blue indicates a decrease > 0.2.

Figure S13. Comparative PARCH analysis of Eda near acetylation sites K24 and K25.

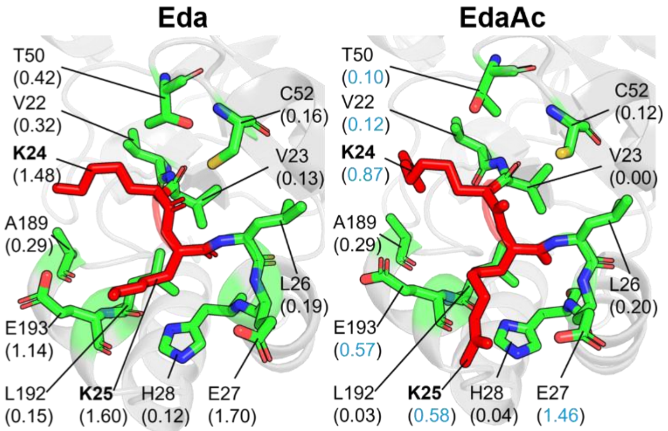

PARCH values of unmodified (left) and acetylated (right) states. Residues are labeled with their identities and PARCH values in parentheses. Red value indicates a PARCH value increase > 0.2 upon acetylation; blue indicates a decrease > 0.2.

Figure S14. Comparative PARCH analysis of Adk near acetylation sites K136, K141 and K145.

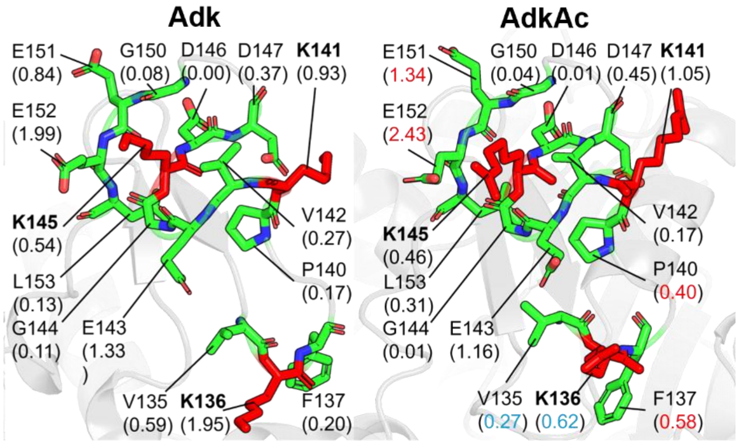

PARCH values of unmodified (left) and acetylated (right) states. Residues are labeled with their identities and PARCH values in parentheses. Red value indicates a PARCH value increase > 0.2 upon acetylation; blue indicates a decrease > 0.2.

Figure S15. Comparative PARCH analysis of Adk near acetylation site K157.

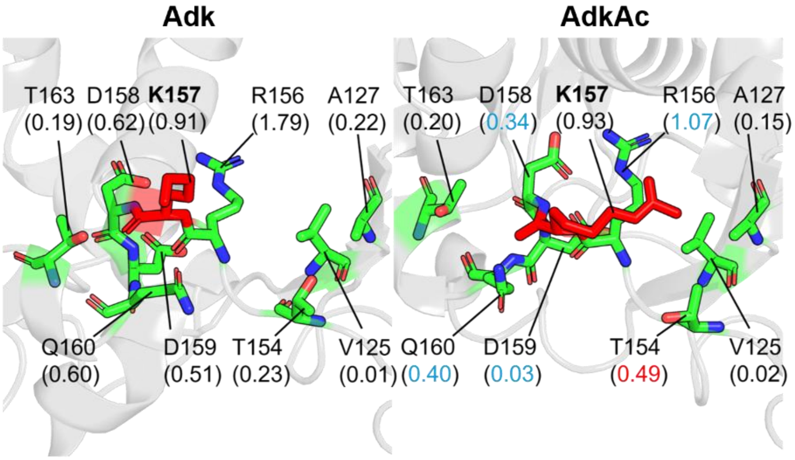

PARCH values of unmodified (left) and acetylated (right) states. Residues are labeled with their identities and PARCH values in parentheses. Red value indicates a PARCH value increase > 0.2 upon acetylation; blue indicates a decrease > 0.2.

Figure S16. Comparative PARCH analysis of Adk near acetylation sites K192 andK211.

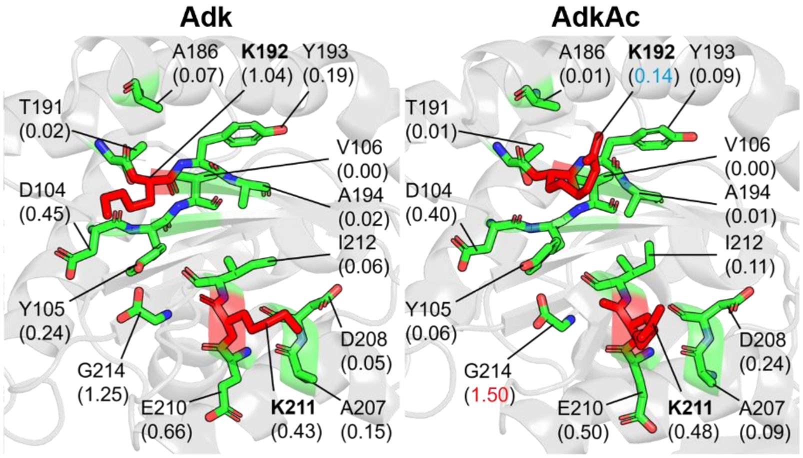

PARCH values of unmodified (left) and acetylated (right) states. Residues are labeled with their identities and PARCH values in parentheses. Red value indicates a PARCH value increase > 0.2 upon acetylation; blue indicates a decrease > 0.2.

Figure S17. Comparative PARCH analysis of pfkA near acetylation site K317.

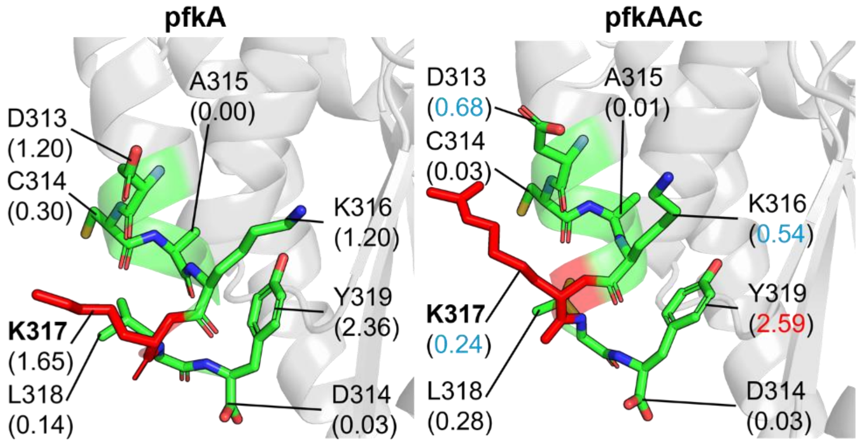

PARCH values of unmodified (left) and acetylated (right) states. Residues are labeled with their identities and PARCH values in parentheses. Red value indicates a PARCH value increase > 0.2 upon acetylation; blue indicates a decrease > 0.2.

Figure S18. Comparative PARCH analysis of PgmA near acetylation sites K17 and K99.

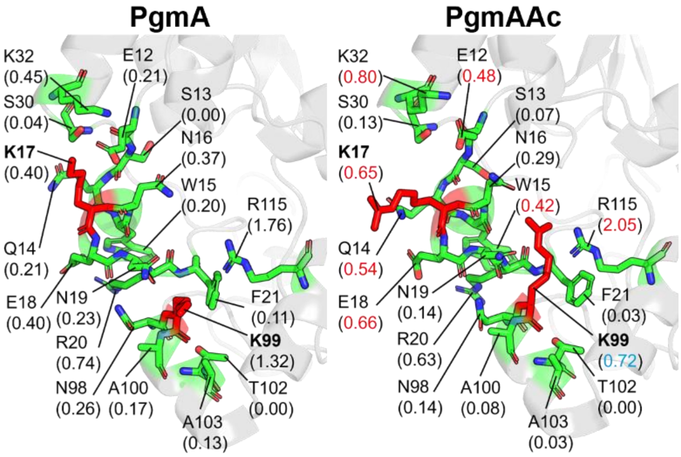

PARCH values of unmodified (left) and acetylated (right) states. Residues are labeled with their identities and PARCH values in parentheses. Red value indicates a PARCH value increase > 0.2 upon acetylation; blue indicates a decrease > 0.2.

Figure S19. Comparative PARCH analysis of PgmA near acetylation sites K85, K141 and K145.

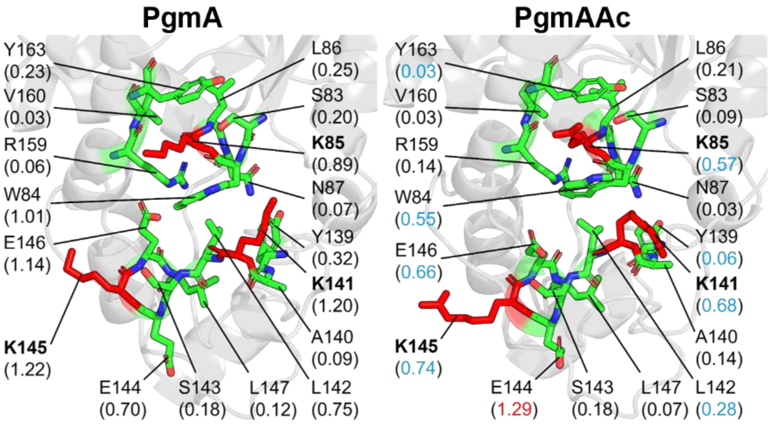

PARCH values of unmodified (left) and acetylated (right) states. Residues are labeled with their identities and PARCH values in parentheses. Red value indicates a PARCH value increase > 0.2 upon acetylation; blue indicates a decrease > 0.2.

Figure S20. Comparative PARCH analysis of GapA near acetylation site K61.

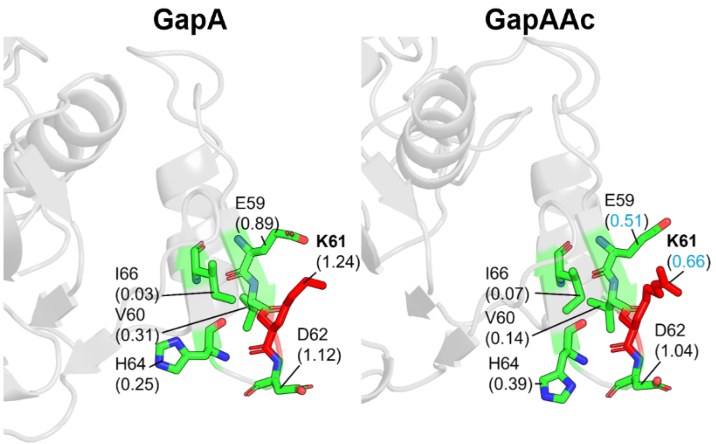

PARCH values of unmodified (left) and acetylated (right) states. Residues are labeled with their identities and PARCH values in parentheses. Red value indicates a PARCH value increase > 0.2 upon acetylation; blue indicates a decrease > 0.2.

Figure S21. Comparative PARCH analysis of GapA near acetylation site K124.

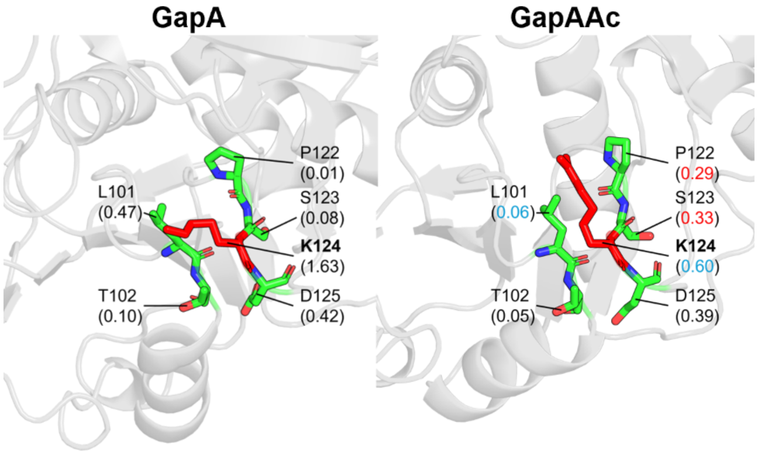

PARCH values of unmodified (left) and acetylated (right) states. Residues are labeled with their identities and PARCH values in parentheses. Red value indicates a PARCH value increase > 0.2 upon acetylation; blue indicates a decrease > 0.2

Figure S22. Comparative PARCH analysis of GapA near acetylation sites K132 and K138.

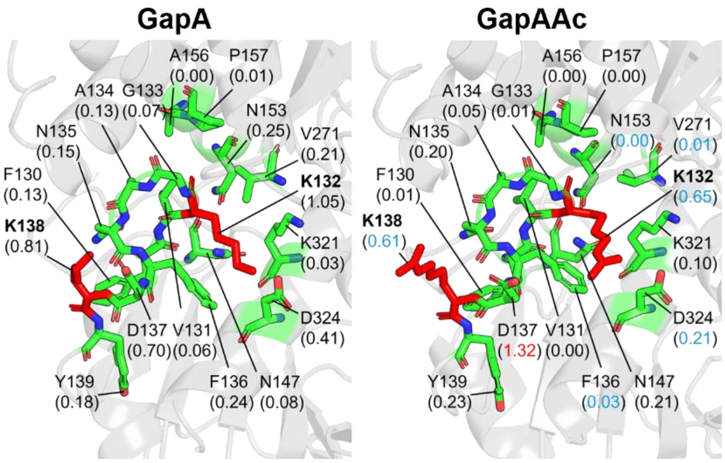

PARCH values of unmodified (left) and acetylated (right) states. Residues are labeled with their identities and PARCH values in parentheses. Red value indicates a PARCH value increase > 0.2 upon acetylation; blue indicates a decrease > 0.2

Figure S23. Comparative PARCH analysis of GapA near acetylation sites K184 and K192.

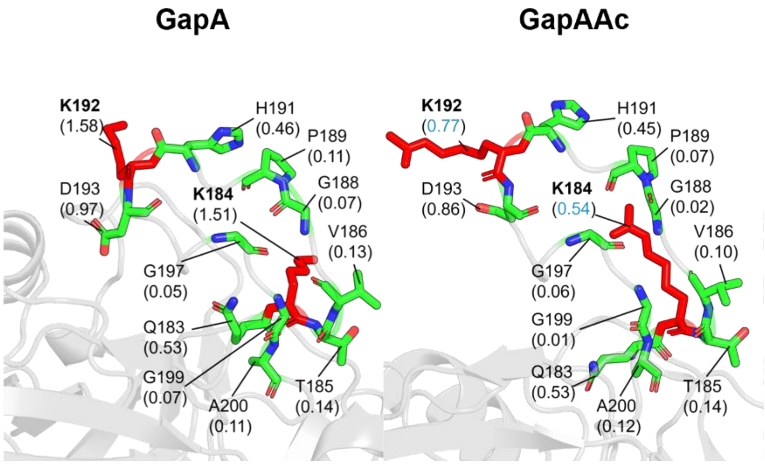

PARCH values of unmodified (left) and acetylated (right) states. Residues are labeled with their identities and PARCH values in parentheses. Red value indicates a PARCH value increase > 0.2 upon acetylation; blue indicates a decrease > 0.2

Figure S24. Comparative PARCH analysis of CYB5B near monomethylation site K382.

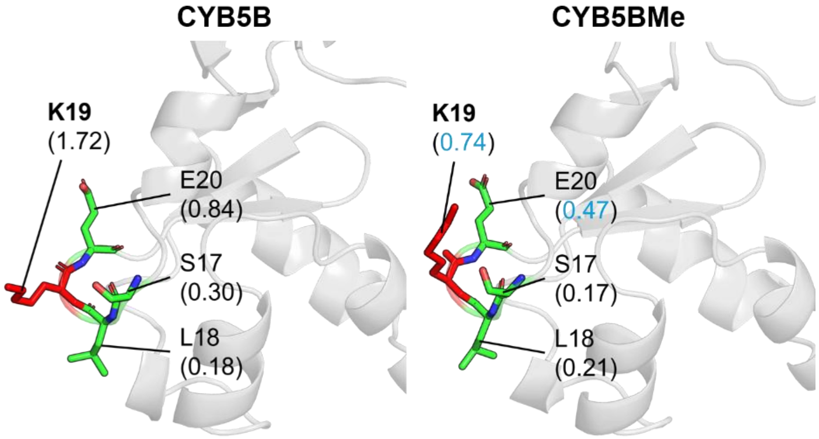

PARCH values of unmodified (left) and monomethylated (right) states. Residues are labeled with their identities and PARCH values in parentheses. Red value indicates a PARCH value increase > 0.2 upon methylation; blue indicates a decrease > 0.2.

Figure S25. Comparative PARCH analysis of PP1G near monomethylation site R36.

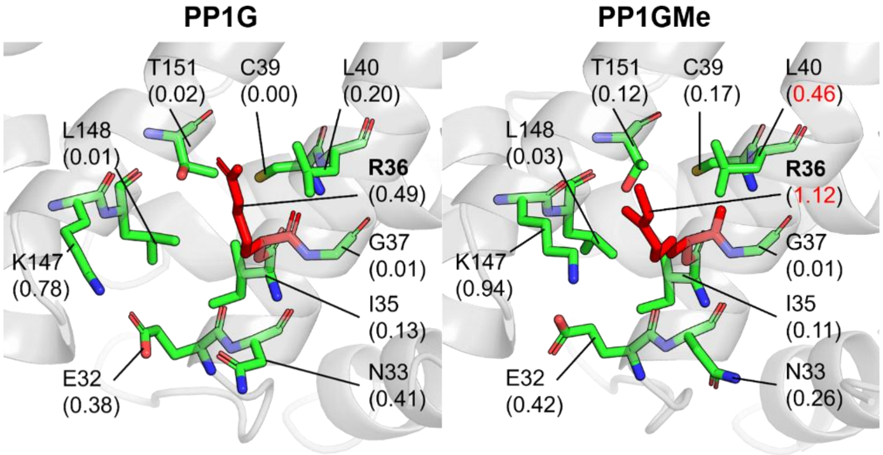

PARCH values of unmodified (left) and monomethylated (right) states. Residues are labeled with their identities and PARCH values in parentheses. Red value indicates a PARCH value increase > 0.2 upon methylation; blue indicates a decrease > 0.2.

Figure S26. Comparative PARCH analysis of NDKA near monomethylation site R6.

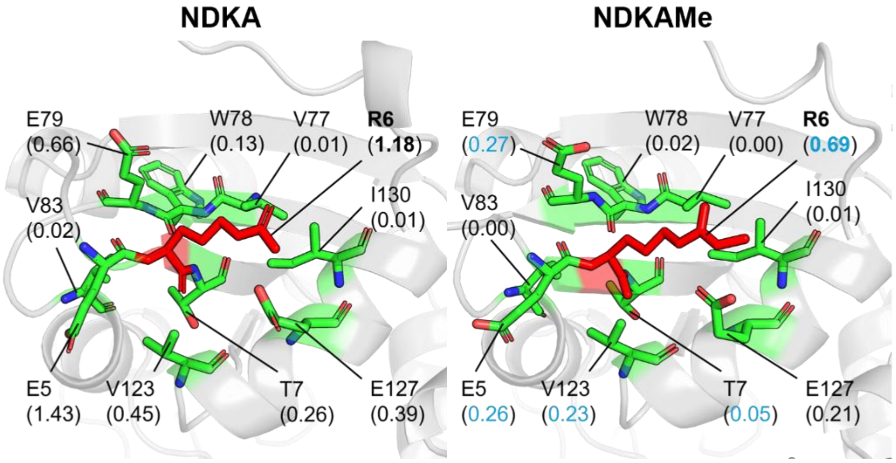

PARCH values of unmodified (left) and monomethylated (right) states. Residues are labeled with their identities and PARCH values in parentheses. Red value indicates a PARCH value increase > 0.2 upon methylation; blue indicates a decrease > 0.2.

Figure S27. Comparative PARCH analysis of NDKA near monomethylation site R58.

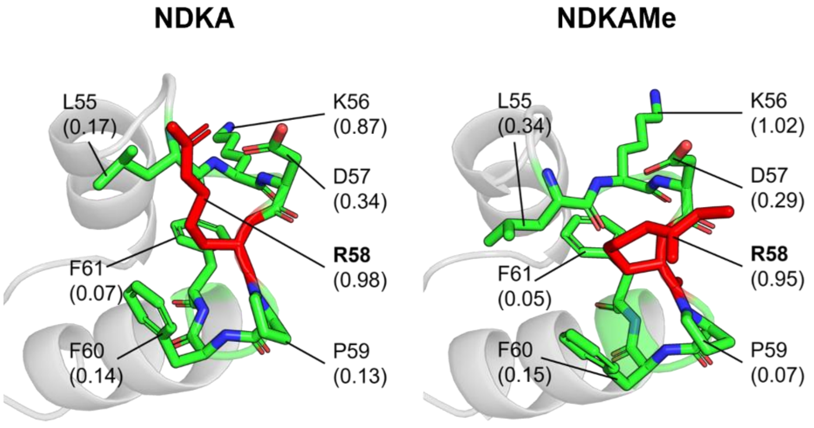

PARCH values of unmodified (left) and monomethylated (right) states. Residues are labeled with their identities and PARCH values in parentheses. Red value indicates a PARCH value increase > 0.2 upon methylation; blue indicates a decrease > 0.2.

Figure S28. Comparative PARCH analysis of p53 near monomethylation site K382.

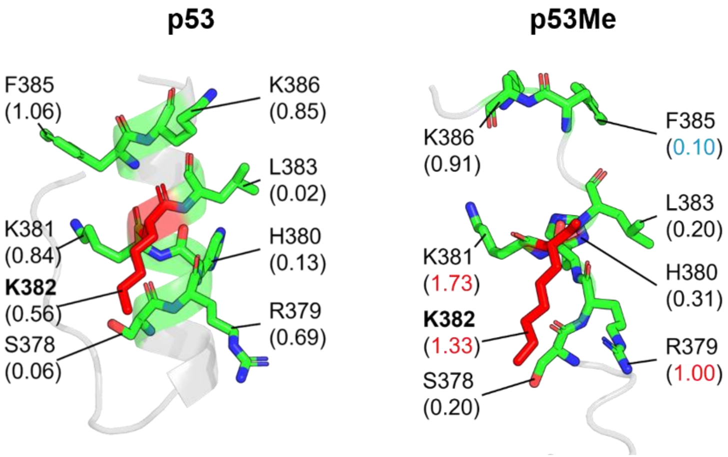

PARCH values of unmodified (left) and monomethylated (right) states. Residues are labeled with their identities and PARCH values in parentheses. Red value indicates a PARCH value increase > 0.2 upon methylation; blue indicates a decrease > 0.2.
